# Current Trends in Presbyopia Correction—A Comprehensive Review

**DOI:** 10.3390/jcm15010215

**Published:** 2025-12-27

**Authors:** Ewelina Trojacka, Joanna Przybek-Skrzypecka, Janusz Skrzypecki, Jacek P. Szaflik, Justyna Izdebska

**Affiliations:** 1 Center of Ocular Microsurgery, Professor Jerzy Szaflik’s Clinic in Warsaw, 00-215 Warsaw, Poland; 2 SPKSO Ophthalmic University Hospital in Warsaw, 03-709 Warsaw, Poland; 3 Department of Ophthalmology, Medical University of Warsaw, 02-091 Warsaw, Poland; 4 Department of Experimental Physiology and Pathophysiology, Medical University of Warsaw, 02-091 Warsaw, Poland

**Keywords:** accommodation loss, presbyopia treatment, Presbyond, refractive lens exchange

## Abstract

Presbyopia is a physiological phenomenon and one of the leading factors contributing to decreased near visual acuity. The prevalence of presbyopia, its social and economic consequences and the prolongation of human life place the correction of presbyopia among the top challenges in modern ophthalmology. Despite the numerous methods currently available for correcting presbyopia, there is still no ideal technique that, by restoring the eye’s age-related loss of physiological accommodation, would provide long-term effectiveness without adverse effects. This article offers an overview of the existing knowledge on the etiology of presbyopia and the available methods of its correction, with particular emphasis on refractive surgery techniques.

## 1. Introduction

Presbyopia represents a progressive loss of near vision resulting from the age-related decrease in accommodation amplitude [1].

In adolescence, the human eye has an accommodation range of about 13 D, which decreases to around 6 D at the age of 40 and 2 D by the age of 50 [1].

According to Donders’ curve, the rate of accommodation loss is about 0.25 D per year [1]. Thus, by the age of around 60, the average eye has only 1 D of accommodation left [1].

The onset of presbyopia is typically observed around the ages of 40–45, slightly more frequently and earlier in women than in men [2,3].

Earlier onset of presbyopia has been observed in populations living in equatorial regions—Central and South America—likely due to increased lens degeneration caused by prolonged exposure to high levels of ultraviolet radiation [4,5,6,7].

Presbyopia is widespread in society, affecting approximately 24% of the global population [8]. In 2015, presbyopia affected about 1.8 billion people across the world, with 826 million unable to obtain adequate optical correction [8].

According to estimates, by 2030, the population of presbyopes will reach 2.1 billion, which is linked to the global increase in the projected life expectancy of societies [8].

Presbyopia is a physiological phenomenon that occurs in every human being and develops in both emmetropic and ametropic eyes. 

Hyperopic individuals, even those with undiagnosed mild hyperopia, will experience presbyopic symptoms earlier than those without a refractive error [3,9]. In contrast, in myopic eyes, functional presbyopia becomes noticeable later than in emmetropic eyes [8,10].

In addition to the deterioration of near vision acuity, presbyopia symptoms also include nonspecific complaints such as double vision, tearing, eye fatigue and headaches [11].

Presbyopia, a leading cause of near vision impairment, is a growing public health, social and economic problem worldwide [12].

The lack of access to adequate presbyopia correction is primarily a problem in developing countries [13,14]. Research shows that in these countries, presbyopia correction through glasses is available to only 6–45% of patients [9,12,15,16,17]. In contrast, in developed countries, widespread access to corrective eyewear, such as “reading glasses,” limits the use of alternative treatment methods [18].

Uncorrected or improperly corrected presbyopia, as well as dependence on optical aids, significantly leads to a decline in quality of life and functional productivity in persons aged 40 and older [13,19].

Throughout the United States, presbyopia and its associated decline in productivity significantly impact the gross domestic product, resulting in an estimated economic loss of approximately $25 trillion [20].

The prevalence of presbyopia, its profound implications for daily functioning combined with the extension of the human lifespan and current trends in visual needs, all contribute to the search for correction methods that ensure long-term independence from optical aids. This paper is intended to provide an overview of currently available methods for correcting presbyopia, with particular emphasis on refractive surgery techniques.

## 2. Methodology

This manuscript was prepared as a narrative review, aiming to provide an overview of contemporary methods of presbyopia correction, including optical, pharmacological, corneal, lens-based and scleral approaches. Although narrative reviews do not follow the strict protocols required for systematic reviews, methodological transparency was ensured and is presented below.

### 2.1. Sources of Information and Databases Searched

The literature search was conducted between July 2025 and December 2025. The following electronic databases and search engines were used:

- PubMed/MEDLINE
- Google Scholar
- General Google search engine

Peer-reviewed scientific articles represented the primary sources. Supplementary information—particularly regarding emerging technologies, regulatory approvals and early clinical results, was also obtained from industry reports, conference abstracts, and reputable press releases when peer-reviewed data were unavailable.

### 2.2. Search Strategy and Keywords

Searches were performed using combinations of the following keywords and phrases:

- presbyopia
- presbyopia correction methods
- corneal refractive surgery in presbyopia correction
- refractive lens exchange in presbyopia correction
- surgical methods of presbyopia correction
- accommodative IOLs
- pharmacological treatment of presbyopia
- contact lenses presbyopia
- pseudoaccommodation

Boolean operators (and, or) and filters for English-language publications were applied when appropriate.

### 2.3. Time Frame and Literature Coverage

The search covered publications from 1966 to 2025, with particular emphasis on articles from the last 10 years due to rapid technological advances in corneal and lens-based surgery. Earlier seminal works and classical theoretical publications were included when historically relevant.

### 2.4. Inclusion and Exclusion Approach

Because this is a narrative review, no formal inclusion or exclusion criteria were predefined. Instead, studies and materials were selected based on:

- Relevance to presbyopia mechanisms or treatment modalities,
- Clinical significance and applicability to current practice,
- Quality and clarity of presented data,
- Contribution to understanding of emerging technologies.

Articles were excluded if they were clearly outdated, duplicated, or unrelated to the scope of presbyopia correction.

### 2.5. Review Process

All identified publications were screened manually. Full texts were read and assessed for relevance. Data were extracted and synthesized descriptively, without statistical pooling or meta-analysis. The review was conducted independently by the author.

### 2.6. Reporting Standards

This review did not follow a specific formalized checklist, as it was not designed as a systematic review. Nonetheless, efforts were made to maintain clarity, transparency and comprehensive coverage, in alignment with good practices for narrative reviews.

## 3. Etiology of Presbyopia

The etiology of presbyopia has not been definitively explained.

The development of presbyopia is closely linked to the accommodation process and therefore, the description of its mechanism takes into account hypotheses explaining the phenomenon of eye accommodation.

The most widely accepted hypothesis explaining the phenomenon of eye accommodation and the one most frequently used to define presbyopia, is Helmholtz’s theory [21,22]. According to this theory, accommodation is governed by the biomechanical flexibility of the lens. This property enables the lens to modify its shape during ciliary muscle contraction and subsequent relaxation of ciliary body fibers, resulting in higher refractive power [21,22]. The hardening of the lens, resulting from degenerative changes in its structure—particularly the increased concentration of water-insoluble proteins, that occurs with age—leads to its opacification and reduced flexibility, leading to a gradual decline in the eye’s ability to adjust focus on near objects [21,22].

However, the sophisticated mechanism of ocular accommodation means that Helmholtz’s theory is insufficient to fully explain the mechanism of presbyopia development [23].

In contrast to Helmholtz’s theory, Schachar proposed an alternative explanation, in which the author claims that when the ciliary muscle contracts during accommodation, the tension in the ciliary body fibers at the lens equator rises, along with a simultaneous relaxation of the fibers in its anterior and posterior groups [24,25]. This distribution of forces causes the lens to elongate in its equatorial dimension, decreasing the volume of its peripheral part and increasing the volume of its central part [24,25]. According to Schachar’s theory, the rise in the lens’s focusing ability during accommodation is a result of changes in the lens’s geometry [24,25]. Schachar’s theory explains the phenomenon of presbyopia not as a reduction in the elasticity of the aging lens, but as an increase in its equatorial dimension. Once this dimension reaches a critical value, the lens’s ability to respond to the forces of the ligament apparatus becomes decompensated [24,25,26].

In recent years, a dynamic approach to defining the concept of presbyopia has gained popularity [27,28]. This approach illustrates the onset of presbyopia by a mechanical model describing age-related changes in the eye. According to this model, presbyopia is not an ocular refraction abnormality or a change in accommodative amplitude, but instead a consequence of mechanical and structural changes at both the extracellular and intracellular levels, as well as modifications to the physiological and biochemical processes of the aging eye [27,28,29]. During the aging process of the eye’s connective tissue, changes in its biomechanics occur [30]. Recent studies highlight the role of changes occurring in the ciliary body and ciliary muscle, as well as in the peripheral choroid and vitreous body, in the development of presbyopia [31,32].

## 4. Methods of Correcting Presbyopia

Presbyopia correction methods can be divided into static and dynamic approaches based on the underlying mechanism of its development, and into conservative and surgical interventions, based on the type of intervention applied [33].

Conservative methods include optical aids and pharmacotherapy, while surgical ones involve more invasive procedures [33].

Static approaches, based on the principles of Helmholtz’s accommodation theory, aim to achieve the desired therapeutic effect by providing a constant compensation for the existing accommodation loss at the time the intervention is applied. This implies that the optical power of the eye does not undergo dynamic alterations when focusing on nearby objects [33]. They do not restore the lost accommodative function; however, by adjusting elements of the optical system—such as the cornea or lens—through creating multifocality or modifying asphericity, they improve pseudo-accommodation by extending the depth of field [33]. Such actions necessitate the acceptance of a compromise, which, on the one hand, improves near vision acuity, but on the other hand, often leads to a deterioration in distance vision, reduced binocular vision function and a weakening of stereopsis [33].

The primary goal of presbyopia correction, ensuring long-term results, is to restore the lost accommodation ability. This principle forms the foundation of dynamic correction methods for presbyopia, which seek to reinstate the eye’s capacity to actively adjust its optical power [33].

To enhance the decision-making algorithm for selecting the most optimal treatment method for patients with presbyopia, the concept of Dysfunctional Lens Syndrome (DLS) was introduced. DLS was first characterized by George Waring IV and his collaborators, and represents a specific gradation of changes occurring in the aging lens [34].

- DLS I—Stiffening lens—presbyopia
- DLS II—Reduced contrast sensitivity, elevated higher-order aberrations and light scattering that diminishes twilight vision—early cataract
- DLS III—Lens opacification impairing daily life activities—cataract

A candidate for presbyopia correction is an individual in stages I and II of DLS. In stage III, the only appropriate intervention is cataract surgery [34].

### 4.1. Optical Methods in Presbyopia Correction

#### 4.1.1. Spectacles

The use of reading glasses—single-vision lenses intended for near tasks—is the oldest and most commonly available method for correcting presbyopia. They supplement the lost optical power of the eye [18,35].

In eyes with existing refractive errors, this method requires the use of an additional pair of glasses, while in emmetropic eyes, it leads to dependency on optical aids. Bifocal and progressive glasses combine corrective lenses for existing refractive errors with an additional near correction to compensate for the lost accommodation range [18,35]. Both types offer the convenience of correcting simultaneously distance and near vision in one pair of glasses.

One drawback is image instability at the junction between the lenses in bifocals and in the peripheral areas of progressive lenses. This can hinder adaptation to this type of correction and increase the risk of falls [18,35,36].

#### 4.1.2. Contact Lenses

Presbyopia can also be corrected using soft contact lenses as well as rigid gas-permeable devices option [35,36,37,38].

Recommended models of contact lens use include:

- Combination of contact lenses aided by glasses

Contact lenses that correct the original refractive error combined with glasses, that add a near correction.

This combination ensures optimal clarity of vision at both distant and near ranges.

It is not very comfortable due to the need for using two types of optical aids.

- Monovision with contact lenses

Monovision involves creating anisometropia. In conventional monovision one—dominant eye is adjusted for distance vision, while the non-dominant eye is corrected for near tasks [39]. In crossed monovision both eyes are corrected in reverse [40].

This approach enables clear vision at both near and far distances without requiring extra optical devices.

The level of anisometropia is controversial and depends heavily on the patient’s visual requirements, age, individual tolerance and lifestyle. Typically, addition in the non-dominant eye should be between +1.5 to +2.5 Dsph. However, achieving optimal binocular visual acuity is accompanied by deterioration of binocular vision parameters, such as contrast sensitivity and stereopsis [39,40,41,42].

- Multifocal contact lenses

Multifocal contact lenses have a refractive design that ensures clear vision at multiple distances: far, intermediate and near [43].

These lenses offer a convenient solution for correcting presbyopia without the need for multiple optical aids.

Successful outcomes largely depend on proper fitting and lens centration, pupil width, tolerance to blur and neuroadaptation. Additionally, their use may be linked to a decrease in night vision quality due to positive dysphotopsias, such as glare and halos [43,44,45].

### 4.2. Pharmacotherapy for Presbyopia

Pharmacotherapy used to alleviate presbyopia employs two mechanisms:

- induction of miosis and the creation of a pinhole effect, both of which lead to an increased depth of focus,
- structural lens alterations, that improve its elasticity [46,47].

Substances that induce miosis and are used in the pharmacological treatment of presbyopia include drugs from the group of muscarinic receptor agonists—pilocarpine, carbachol, aceclidine, as well as a non-selective adrenergic receptor blocker—phentolamine or combined agents [46]. All the above mentioned are administered only to one-non-dominant eye [46,48].

A representative of lens-softening agents is the choline ester of lipoic acid [49].

#### 4.2.1. Muscarinic Receptor Agonists

##### Pilocarpine

Pilocarpine is a muscarinic receptor agonist that specifically targets the M3 receptor subtype [50].

Stimulation of muscarinic receptors by pilocarpine leads to an increase in intracellular calcium concentration in the fibers of both the pupillary sphincter and the ciliary muscles, causing their contraction. As a result, miosis and accommodative effort are induced [50].

In presbyopia management, the effectiveness and safety of pilocarpine have been confirmed for solutions at two concentrations: 1.25% and 0.4% [51,52,53].

The efficacy and risk profile of the 1.25% pilocarpine eye drops were analyzed in the GEMINI and the VIRGO studies, while the 0.4% solution was evaluated in the NEAR study [51,52,53].

In the GEMINI study, 1.25% pilocarpine hydrochloride solution was administered once daily for 30 days, while in the VIRGO study, twice daily for 14 days [51,52].

In the NEAR study, 0.4% pilocarpine hydrochloride solution was administered twice daily for 2 weeks [53].

In each of the aforementioned studies, the use of pilocarpine resulted in a significant enhancement of near visual acuity with distance correction (DCNVA) by a minimum of 3 lines, accompanied by a concurrent reduction in binocular corrected distance visual acuity (CDVA) not exceeding 5 letters (in the GEMINI and VIRGO studies) or 1 line (in the NEAR study) [51,52,53]. This is consistent with the main endpoint defined by the FDA for all clinical trials of presbyopia-correcting eye drops [51,52,53,54].

The most commonly reported adverse effects during studies of pilocarpine use were: headaches, conjunctival hyperemia, blurred vision and eye pain upon drop administration. These symptoms were reported by patients receiving pilocarpine at concentrations of 1.25% and 0.4% with the following frequencies, respectively: headaches: 8.8–14.9% and 6.8%, ocular redness: 5.1% and 1.6%, dim vision: 4.4–4.5% and 3.6%, eye pain: 2.6–4.3% and 5.8% [51,52,53].

Both 1.25% and 0.4% pilocarpine solutions, under the trade names Vuity and Qlosi, have received FDA approval for the pharmacological management of presbyopia [50,52,54,55].

##### Carbachol

Carbachol is a parasympathomimetic drug that stimulates both nicotinic and muscarinic receptors [50].

Similarly to pilocarpine, carbachol causes contraction of the pupillary sphincter and ciliary muscles. However, because it non-selectively stimulates all muscarinic receptor subtypes, carbachol produces a greater extent and longer duration of muscle contraction compared to pilocarpine [50].

It also requires less frequent dosing than pilocarpine [50].

The effectiveness of carbachol in alleviating the symptoms of presbyopia was evaluated in the VIVID study. This multi-center randomized, double-masked three-arm crossover study, aimed at assessing the safety and efficacy of fixed-dose combinations of carbachol and brimonidine tartrate (Brimochol, Brimochol F, Tenpoint Therapeutics, London, UK) versus a similarly formulated preservative-free carbachol, enrolled 85 participants with emmetropic phakic or pseudophakic presbyopia, aging between 45 and 80 years [56]. The primary endpoint of this study—3 lines enhancement of binocular near visual acuity (NVA) without a reduction in one line in distance visual acuity (DVA), was achieved in all three investigated groups showing a minimum responder rate of 83% at 1 h, 82% at 3 h, 52% at 7 h and 35% at 9 h post-administration [56,57].

Abdelkader et al. in a double-masked randomized prospective controlled clinical trial evaluated the effectiveness of carbachol and brimonidine in alleviating the symptoms of presbyopia [48]. The study included a group of 10 emmetropic presbyopes aged 42 to 58 years. Each participant received, at weekly intervals in the weaker eye, separate or combined 3% carbachol and 0.2% brimonidine, as well as 3% carbachol alone and 0.2% brimonidine alone [48]. After administration of 3% carbachol, a notable enhancement in near visual acuity was noted—from a baseline value of J 8.6 ± 1.5 to J 5.5 ± 1.0, J 5.9 ± 0.8, J 7 ± 1.2 and J 7.5 ± 1.0 at one, two, four and eight hours after administration, respectively [48].

##### Aceclidine

Aceclidine is a selective parasympathomimetic compound that functions as a modulator of M3 muscarinic receptor subtypes and as an agonist at M1 receptor subtypes [50].

It primarily targets the nerve endings of the pupillary sphincter muscle, with minimal stimulation of the ciliary muscle [50,58,59].

The effectiveness and safety of aceclidine in managing presbyopia have been confirmed in the CLARITY study [59,60,61].

CLARITY is a Phase 3, multicenter, double-blind, randomized, controlled study designed to evaluate efficacy and safety. CLARITY 1 and CLARITY 2 assessed the LNZ 100 (aceclidine 1.44%) efficacy and safety in 466 participants who received once-daily dosing for 42 days, while CLARITY 3 examined the enduring safety of the formulation over a 6-month period of once-daily use in 217 patients [60].

The CLARITY 1 trial compared the efficacy and safety of LNZ100 with brimonidine 0.08%, while CLARITY 2 compared LNZ100 with placebo. The primary endpoint for both studies was the proportion of patients who achieved an advancement of three or more lines from baseline in DCNVA, without a loss of one line (5 letters or more) in CDVA, measured three hours after treatment [59,60,61]. Both the CLARITY 1 and 2 studies, met the primary endpoint [59,60,61].

The LNZ 100 resulted in steady enhancement in near vision across both studies. In the CLARITY 1 study, 72% of patients and in CLARITY 2, 71% of patients who received a 1.44% aceclidine solution once daily, experienced an improvement in DCNVA by at least 3 lines within 30 min of administration. This effect persisted for up to 10 h in 27% of participants in CLARITY 1 and 40% in CLARITY 2 study [59,60,61]. The formulation showed good tolerability, with no significant treatment-associated adverse events reported in any of the three trials. The most frequently observed side effects were instillation site irritation (20%), blurred vision (16%) and headache (13%) [59,60,61]. Less frequently, some participants developed conjunctival hyperemia (8%). Most unfavorable events were mild, short-lived and resolved without intervention [59,60,61].

When administered as eye drops, aceclidine acts quickly, with near vision improving within 30 min and effects lasting up to 10 h [59].

Moreover, due to its selective stimulation of the pupillary sphincter muscle, aceclidine increases depth of focus without the undesired worsening of distance vision, that is typically caused by myopic shift following the use of general parasympathomimetics [59].

In 2025, a 1.44% aceclidine formulation, marketed under the brand name VIZZ, received FDA approval for the pharmacological therapy of presbyopia [62].

##### Side Effects of Parasympathomimetic Drugs

Besides the mild side effects mentioned above, parasympathomimetic drugs, by acting not only on the pupillary sphincter muscle but also on the ciliary muscle, cause a myopic shift that consequently worsens DVA [50].

Furthermore, the transfer of contractile force from the ciliary muscle through the vitreous body may potentially cause vitreoretinal traction, retinal tears and detachment of the retina [50].

Retinal detachment and vitreoretinal traction isolated cases have been reported in patients using 1.25% pilocarpine solution. However, so far, there are no reports of vitreoretinal complications in patients treated with 0.4% pilocarpine [50,63,64,65,66,67].

Elhusseiny et al. have published an evaluation of the likelihood of retinal detachment in patients over 40 years old treated with 1.25% (or other concentrations) pilocarpine [68].

The following summarizes the observed results of retinal detachment risk in both—the study and control groups: after 3 months of pilocarpine use—0.53% and 0.25%, after 6 months—0.6% and 0.31% and after 12 months—0.78% and 0.33%, respectively [68]. The authors observed that the risk of retinal detachment in the study group was 3.14 times that of the control group [68].

Chronic use of muscarinic receptor agonists may lead to stimulation of the uveal tract and induction of inflammation, resulting in pigment dispersion and the formation of posterior synechiae [46,50].

#### 4.2.2. Non-Selective Adrenergic Receptor Blocker Phentolamine

Phentolamine functions as a non-selective alpha-adrenergic receptor antagonist, targeting both alpha-1 and alpha-2 subtypes. The mechanism of action involves specific inhibition of alpha-1 receptors in the radial iris dilator muscles, without impacting the ciliary muscle.

Its direct effect is the relaxation of the pupillary dilator muscle, which indirectly induces miosis and increases depth of focus—leading to potential improvement in NVA [50].

The effectiveness and safety profile of 0.75% phentolamine (Phentolamine Ophthalmic Solution—POS) in alleviating presbyopia symptoms have been confirmed in the VEGA studies [69,70].

In VEGA-1, a Phase 2, randomized, multi-center, placebo-controlled, double-blind clinical trial, participants with presbyopia and DCNVA of 20/50 or worse were randomly assigned to receive POS or placebo for 3–4 evening doses [69]. The morning following the last evening dose, pupil diameter (PD) under mesopic and photopic lighting conditions and visual acuity were assessed at multiple time points. The average baseline PD was 4.5 mm (photopic) and 5.0 mm (mesopic) in the POS group and 4.3 mm (photopic) and 5.1 mm (mesopic) in the placebo [69]. POS treatment resulted in a pupillary response similar to that seen with placebo and produced a statistically significant improvement in the proportion of presbyopic subjects achieving ≥3-line gain in DCNVA compared with placebo (30% vs. 14%), without any loss of best-corrected distance visual acuity (BCDVA) at 12 h, while preserving a dynamic pupillary response [69]. Result of such therapy was a durable improvement in near vision with a favorable safety profile [62].

POS’s mechanism uniquely reduces pupil size by inhibiting the iris dilator muscle, which preserves dynamic pupillary responses in light and dark conditions, in contrast to miotic eyedrops that contract the sphincter muscle with a more limited pupillary response [69,70].

VEGA-3 serves as the second Phase 3 clinical trial examining the safety and efficacy profile of POS in subjects with presbyopia [71]. This Phase 3 study, conducted across 40 U.S. sites, was multicenter, randomized, double-blind and placebo-controlled, with 545 participants enrolled [71]. Subjects were assigned in a 3:2 ratio to receive evening doses of POS or placebo once daily. At 1 h post-dose on day 1, 20.6% of POS-treated participants attained a ≥15-letter (≥3-line) improvement in DCNVA versus 6.1% of those receiving placebo [71]. On day 8, at 12 h post-administration, 27.2% of POS participants achieved the same improvement in DCNVA with less than a 5-letter decrease in BCDVA, versus 11.5% in those receiving placebo [71]. Following 6 weeks of administration, the study showed no evidence of tachyphylaxis [71]. The safety profile of POS in this study aligned with previous trials, showing no emergence of new safety concerns and no serious adverse events attributed to treatment [71]. Conjunctival hyperemia, instillation site irritation and dysgeusia were the most common (≥5%) treatment-emergent adverse events, and the majority were mild in severity [71]. Throughout the study, the occurrence of headache was low, affecting 2.6% of subjects [71].

All the above-mentioned substances owe their effectiveness in alleviating presbyopia symptoms to the induction of miosis, creation a pinhole-induced increase in depth of focus.

Their effectiveness in treating presbyopia is limited by the pharmacokinetics of the active substance used and associated adverse events [50].

To limit the side effects of parasympathomimetics or to prolong their duration of action, combinations with other substances have been introduced [50].

#### 4.2.3. Combined Medications

The combination of 0.247% pilocarpine with 0.78% phenylephrine, marketed as FovTears, was intended to reduce the formation of posterior synechiae and improve visual acuity under mesopic conditions [50,72].

An analysis of a series of clinical cases showed an improvement in NVA by at least 2 lines in patients using FovTears. Moreover within 2 h of administration of this agent, the pupil demonstrated no significant response under photopic (bright light) conditions and only a minimal myopic shift of about 0.17 diopters [50,72].

To limit the induction of inflammation in the uveal tract during pilocarpine therapy, attempts have been made to simultaneously use a nonsteroidal anti-inflammatory drug (NSAID) [72,73].

According to reports by Benozzi et al., the combination of 1% pilocarpine with 0.1% diclofenac provides effective presbyopia therapy by improving NVA without worsening distance or intermediate visual acuity, prolonging the duration of action and reducing the risk of inflammatory responses associated with pigment dispersion, persistent miosis and posterior adhesion formation [74]. This effect is explained by diclofenac’s inhibition of cyclooxygenase and the subsequent production of prostaglandins [73,74].

The combination of carbachol with brimonidine, marketed as Brimochol, was evaluated in the VIVID and BRIO studies [50,56,75,76,77,78].

In VIVID study Brimochol and Brimochol PF achieved the FDA-designated primary endpoint for all studies evaluating presbyopia-correcting eye drops with a minimum responder rate of 83%, 82%, 52% and 35%, respectively, 1, 3, 7 and 9 h after administration [56,57].

Two Phase III pivotal studies, which evaluated the safety, tolerability and efficacy of the formulation, BRIO-I and BRIO-II, have been completed for Brimochol PF [75,76,77,78].

In the first of the two Phase 3 trials, BRIO-I, Brimochol successfully fulfilled all primary and secondary outcome measures [75,76]. The study administered the combination therapy to 182 emmetropic patients, between 45 and 80 years of age, at 15 sites throughout the United States [75,76]. Each subject initially received a single dose of Brimochol PF, carbachol monotherapy, or brimonidine monotherapy, with crossover to the other two arms at later study visits [75,76]. Brimochol PF outperformed carbachol and brimonidine monotherapies at 1, 2, 4, and 6 h post-dose in the percentage of patients reaching the predefined primary endpoint [75,76]. Among participants receiving Brimochol PF, 85% achieved 20/40 near vision at 1 h, 65% at 6 h, and 56% at 8 h following administration [75,76]. At 10 h after administration, approximately half of the subjects maintained 20/40 near visual acuity [76]. Distance vision remained stable across all three study arms in BRIO-I [76]. Brimochol PF treatment resulted in a small yet statistically significant gain in uncorrected distance vision across all assessed time points through 8 h [76]. The study observed no serious adverse events related to treatment and no subjects withdrew because of adverse events [76]. Eye irritation immediately following instillation and headache were the most commonly reported adverse effects in the Brimochol PF group, with irritation reported in 14% of patients [76].

BRIO-II, the second Phase 3 study, was evaluating both the efficacy of Brimochol PF compared with vehicle and its safety with once-daily administration over 12 months [78,79,80]. In this multicenter, randomized, double masked study, statistically significant improvements in near visual acuity versus vehicle were observed at all time points up to 8 h, confirming that Brimochol PF met its primary endpoint [78,79]. Significant decreases in pupil diameter, both clinically and statistically, were observed with Brimochol PF at every time point [78]. Over a 12-month daily administration period, Brimochol PF demonstrated good tolerability and no treatment-related serious adverse events [78].

Brimochol PF’s New Drug Application (NDA) for presbyopia has been submitted to and accepted by the Food and Drug Administration (FDA), with a Prescription Drug User Fee Act (PDUFA) date scheduled for 28 January 2026 [81].

Brimonidine inhibits the action of the pupillary dilator muscle and constricts blood vessels, thereby sustaining carbachol’s effectiveness in inducing miosis [50].

Compared to carbachol alone, Brimochol provides extended efficacy, with near visual acuity gains sustained for up to 8 h and less conjunctival hyperemia [77,78].

Brimochol PF’s once-daily regimen and combined mechanism of action, aim to address the limitations of pilocarpine, which requires multiple daily doses and is associated with headaches and hyperemia [76,78,82].

#### 4.2.4. Drugs That Reduce Lens Stiffness

A lens-softening drug is the choline ester of lipoic acid [49].

Its effectiveness in pharmacotherapy of presbyopia was evaluated under the name of UNR844.

In a biochemically young crystalline lens, free sulfhydryl groups of amino acids that make up the structural proteins of lens fibers allow free movement of cytosol and alterations in lens morphology. Due to oxidative degradation occurring with age, sulfhydryl groups form disulfide bridges, which significantly limit cytosol mobility and the potential for lens shape change.

When administered topically to the eye, the choline ester of lipoic acid undergoes chemical conversion and reaches the crystalline lens, where as a dihydrolipoic acid causes the breakdown of disulfide bonds [49].

The efficacy and safety of UNR844 in alleviating presbyopia symptoms were evaluated in a prospective, randomized, multicenter, double-masked trial, with findings published in 2021.

After 91 days of treatment, improvement in binocular DCNVA was observed in 53.1% of participants in the treatment cohort and in 21.7% of subjects in the control cohort. The treatment effects persisted for 7 months after discontinuation of the therapy [49,83]. These results were not confirmed in the second phase of the READER study, what has currently halted further investigation of this active substance [84].

### 4.3. Refractive Surgery in Presbyopia Correction

Refractive surgery for presbyopia encompasses a range of methods aimed at modifying components of the eye’s optical system or those potentially involved in the accommodation process, such as the cornea, lens and sclera. These procedures aim to restore or enhance the eye’s accommodative capacity.

Each of the existing surgical methods for correcting presbyopia is associated with certain undesirable side effects and the key to success lies in proper patient selection, considering their expectations, available methods and their respective limitations [85].

#### 4.3.1. Corneal Refractive Surgery in Presbyopia Correction

Corneal refractive surgery for presbyopia involves procedures that reshape the cornea to enhance near focusing ability, often utilizing monovision effect, the creation of multifocality and the modification of spherical aberrations. These procedures aim to optimize visual performance for both near and distance focusing.

The main techniques used in surgical corneal approaches for presbyopia correction include:

- corneal inlays implantation,
- conductive keratoplasty,
- laser surgery [33].

##### Corneal Inlays Implantation

Corneal curvature modification for ametropia correction was first introduced by Jose Barraquer in the 1940s and corneal inlays for presbyopia have been investigated since 1949 [86,87]. Modern corneal inlays are implanted in the non-dominant eye at varying stromal depths to enhance near vision by modifying corneal optics [86].

In the management of presbyopia, both synthetic and allogenic corneal implants have been utilized to restore near vision, each with distinct mechanisms of action and safety profiles [86,88].

Synthetic Corneal Inlays

Synthetic inlays initially showed promise but revealed limitations due to biocompatibility, corneal haze and other complications.

Common synthetic inlays include:

- Presbia Flexivue Microlens

A refractive inlay with a bifocal design: a central distance plano zone and a peripheral near-vision zone with a progressive power gradient of +1.25 D to +3.5 D [86,89]. It is implanted in a 300 µm deep corneal pocket created with a femtosecond laser [86]. Clinical outcomes revealed a reduction in one or more lines of CDVA in 60% of patients, with ~10% experiencing >3-line loss [90,91]. Although marketed in select regions, the device is not FDA-cleared for clinical use in the U.S. [92].

- Raindrop Near Vision Inlay

A corneal reshaping inlay implanted at 200 µm depth or beneath a LASIK flap. It creates a hyperprolate corneal shape, inducing multifocality without intrinsic refractive power [86,93,94]. Despite FDA approval in 2016, the Raindrop was discontinued in 2018 due to corneal haze and unsatisfactory outcomes. FDA data reported a 7% explantation rate and corneal haze in 42% of patients after 5 years [86,95,96,97,98].

- KAMRA Inlay

A small-aperture inlay placed at 200–250 µm depth. The pinhole effect improves near and intermediate vision while preserving distance acuity [86,99,100]. Limitations include: decreased stereopsis, corneal haze, hyperopic shift, CDVA loss ≥ 2 lines in 3.4% at 24 months, and 8% explantation rate [98,101,102]. Additional interventions (11%) included repositioning, lamellar rinsing, or supplementary refractive surgery [98,101,102].

Synthetic corneal inlays can fail due to several interrelated mechanisms, including chronic keratocyte irritation secondary to a foreign body response, impaired nutrient transport within the corneal stroma and stromal remodeling or haze that degrades visual quality. Additional challenges arise from difficulties in achieving optimal centration and ensuring adequate biomechanical adaptation. Collectively, these factors account for the absence of FDA-approved synthetic corneal inlays for presbyopia in the United States [86,97].

Allogenic Corneal Inlays

Allogenic inlays are derived from human corneal tissue and aim to mitigate complications associated with synthetic implants [88].

Currently available allogenic inlays include:

- TransForm Corneal Allograft (TCA; Allotex, Boston, MA, USA)

SMILE-derived lenticules, sterilized and shaped with an excimer laser, implanted under a femtosecond laser flap. The available data show, that in 12 emmetropic presbyopic patients, UNVA improved from 0.52 ± 0.14 logMAR to 0.20 logMAR at 6 months, with minimal changes in BCDVA [103].

- PEARL (Presbyopic Allogenic Refractive Lenticule)

SMILE-derived lenticule implanted in a 120 µm intracorneal pocket. Data suggest, that all patients achieved UNVA of J2 and UDVA of 20/20 [104,105].

Allogenic corneal inlays offer several potential advantages over synthetic implants, including a reduced foreign body response, preservation of corneal transparency and effective improvement in near vision. However, they are not without limitations, such as the risk of graft rejection, challenges in achieving precise centration and the need for individualized adjustment of optical power. Current evidence is derived from small patient cohorts and long-term efficacy, and safety have yet to be established [88,104,105,106].

Table 1 provides a comprehensive summary of the characteristics, mechanisms and clinical outcomes of intracorneal implants.

##### Conductive Keratoplasty

Conductive keratoplasty is a procedure that alters the corneal curvature of the non-dominant eye by delivering low-power, high-frequency electrical energy [107].

This energy is applied through a cannula in a circular pattern within optical zones of 6.0, 7.0, or 8.0 mm, following an established nomogram [107]. Throughout the procedure, depending on the case, 8 to 32 applications are made, with the generated heat modifying the cornea’s biomechanics by affecting collagen [107]. As a result, the central cornea becomes steeper, leading to greater refractive power and improved near vision [107].

At 12 months post-procedure, Stahl reported that conductive keratoplasty was effective and safe in emmetropic presbyopes [108]. According to these findings, 90% of patients achieved UNVA at J1 and 100% at least at J3. The procedure resulted in an average gain of 8.7 ± 2.0 lines in UNVA, although uncorrected distance visual acuity (UDVA) decreased by 2.2 ± 2.0 [109].

Unfortunately, the significant instability and regression of results over time make this method less popular for presbyopia correction [109,110]. Conductive keratoplasty, as a “failed procedure” is of historical significance and is no longer used in clinical practice for the correction of presbyopia due to its low effectiveness [110].

##### Refractive Laser Surgery

Corneal refractive laser surgery used in the correction of presbyopia primarily employs techniques such as photorefractive keratectomy (PRK) and LASIK [111,112]. Near vision may be improved via monovision, multifocal ablation profiles and a non-linear aspheric ablation profile with micro-monovison (Presbyond Laser Blended Vision, LBV method).

- Monovision

Corneal refractive laser surgery is commonly used to create monovision, which is among the most popular strategies for presbyopia correction [113].

Corneal ablation is applied to both eyes—dominant and non-dominant—using an excimer laser and creating anisometropia that provides optimal near vision, in line with the principles of monovision. Traditional monovision corrects the dominant eye to emmetropia for distance and the non-dominant eye to myopia for near vision. Crossed monovision reverses the visual roles of the dominant and non-dominant eyes [40,114].

The target refraction remains controversial, with some authors suggesting that anisometropia should be in the range of 2.5 Dsph, while others argue it should not exceed 2.0 Dsph. Similarly to monovision achieved with optical aids, the degree of anisometropia is primarily influenced by the patient’s age, visual requirements, lifestyle and tolerance for aniseikonia [113,115,116].

Anisometropia of 1.75 D or higher, resulting from monovision, can lead to reduced visual acuity at intermediate and distance ranges, lower contrast sensitivity and a loss of stereoscopic vision [115,116,117,118,119].

The results and patient satisfaction following monovision surgery performed on a group of myopic presbyopic patients were presented by Wright et al. and Levinger et al. [118,120]. Wright et al. analyzed the outcomes of refractive photokeratectomy surgery with monovision creation and a target anisometropia of 1.25 D, performed on a group of 21 myopic presbyopic patients [120]. A binocular uncorrected distance visual acuity (UDVA) of 20/25 or better was attained by 95.3% of participants [120]. All patients were able to perform near tasks without reading glasses after surgery [120]. Stereopsis of 40–800 arcseconds and binocular fusion were preserved in all patients [120]. The average patient satisfaction with the procedure was reported at 86% [120].

Levinger et al. prospectively studied 40 myopic presbyopes treated with monovision laser correction and anisometropia of 1 D or greater, evaluating binocular UDVA, stereopsis, contrast sensitivity, including patient satisfaction, before surgery as well as at a 12-month follow-up [118]. 

The following results were presented, respectively, before and 12 months after the procedure:

- binocular UDVA was 0.87 ± 0.2 logMAR and 0.09 ± 0.11 logMAR,
- distance stereopsis was 52 and 142 arcseconds, near stereopsis was 54 and 57 arcseconds,
- patient satisfaction was 41.5 ± 30.4% and 85.2 ± 5.0% [118].

Additionally, the researchers observed a decrease in contrast sensitivity [118].

In another study, Levinger et al. evaluated the outcomes and patient satisfaction following surgery in a group of 100 myopic and 14 hyperopic presbyopes [121]. The follow-up period was 90 days or more after the procedure [121]. Most patients achieved excellent visual outcomes: 79% reached binocular UDVA ≥ 6/7.5 and 97% reached binocular UNVA ≥ J2 [121]. The median near stereopsis was 100 arcseconds. 80% of patients were very satisfied with the surgical results [121].

After surgery, 8% of patients still required glasses for distance and 24% needed glasses for reading [121]. Quality of life related to driving at night and during the day improved in 55% and 69% patients, respectively, while it worsened in 15% and 1% of patients [121]. A comparative outcome analysis between LASIK with monovision creation and refractive lens exchange (RLE) with the Tecnis Symfony multifocal intraocular lens implanted, was carried out by Schallhorn and colleagues [122]. Patients with presbyopia were categorized into four groups based on their initial refractive error: hyperopia, plano presbyopia, low myopia and moderate-high myopia [122].

At the three-month follow-up binocular UDVA ≥ 20/20 was observed, respectively, at rates of 84.7%, 89.4%, 90.5%, 77.5% after LASIK and 90.7%, 85.2%, 89.9%, 84.2%, after RLE [122].

Binocular UNVA of 20/40 or better was achieved by 98.9%, 100%, 96.8% and 95.6% of patients after LASIK, and 90.7%, 98.1%, 95.8%, 95.7% after RLE [122]. A significant difference, in statistical terms, was found by the investigators in post-operative satisfaction favoring LASIK exclusively in patients with moderate to high myopia [122]. In other categories, satisfaction differences were not statistically significant. Furthermore, among myopic patients undergoing RLE, postoperative photic phenomena occurred more frequently than in patients who underwent LASIK [122].

- Multifocal corneal ablation

Multifocal corneal ablation techniques use excimer-laser–based shaping of the cornea to create a multifocal optical profile that provides functional vision at far, intermediate and near distances [123,124]. These approaches were first introduced in the early 1990s and expanded clinically after the evolution of LASIK, and the introduction of femtosecond lasers, which improved flap precision, predictability, and postoperative recovery [119]. Two main optical concepts are applied: central-near and peripheral-near multifocality, each assigning different refractive powers to the central and peripheral cornea [124,125].

In central-near profiles, the central cornea provides near addition, while the peripheral cornea remains optimized for distance vision [124,125,126]. In peripheral-near profiles, a ring-shaped peripheral zone enhances near performance, while central optics remain distance-focused [124,125,126]. These profiles are typically implemented during LASIK and are generally referred to as central presbyLASIK and peripheral presbyLASIK, respectively [124,125]. Central profiles generally require less ablation, induce fewer higher-order aberrations and exhibit reduced dependence on pupil diameter [124,125]. They also align better with the physiological convergence-accommodation reflex and therefore often result in shorter neuroadaptation times [124,125].

Commercially available multifocal ablation platforms include AMO VISX, Technolas SUPRACOR and SCHWIND PresbyMAX, each with specific algorithms, asphericity targets, and distribution of refractive power across the cornea [126].

- AMOVISX

The AMO VISX approach for hyperopia-presbyopia targets patients with hyperopia up to +4.0 Dsph, astigmatism up to −2.0 Dcyl and presbyopia [127].

This procedure employs the AMO VISX STAR S4 excimer laser to perform aspheric ablation for presbyopia alongside LASIK wavefront-guided ablation for underlying refractive errors [126].

In a 12-month follow-up study, 100% of patients who underwent the procedure achieved binocular UNVA at least at the J3 level and binocular UDVA better than 20/25. However, more than two lines of preoperative CDVA were lost in 10% of cases [127].

- SUPRACOR

SUPRACOR Technolas is a technique that involves creating a hyperpositive central zone with a +2.0 Dsph addition within the cornea’s central 3 mm area. The peripheral cornea is targeted for different refractions: around −0.5 Dsph in non-dominant and +0 Dsph in the dominant eye [126,128].

The application of an optimized algorithm decreases aberration induction [128].

Ryan et al. were the first to report clinical outcomes of the SUPRACOR presbyopia-correcting ablation. They found that 91% of patients achieved a binocular UDVA of 0.2 logMAR or better, and the same proportion reached an uncorrected reading performance of N8 or better. Six percent of eyes lost two or more lines of CDVA. Notably, 93% of patients reported complete independence from reading glasses. Although a modest increase in higher-order aberration (HOA) RMS was observed, no clinically significant rise in coma or trefoil was detected [129].

In a 1-year follow-up study, Schlote et al. reported that 87.2% of patients achieved an UNVA better than 0.4 logMAR after SUPRACOR treatment; however, 40% continued to use reading glasses on a daily basis. A loss of two lines of CDVA occurred in 10% of treated eyes [130].

Saib et al. evaluated outcomes using the standard SUPRACOR algorithm combined with micro-monovision. At 1 year, 100% of patients achieved a UDVA of 20/25 and an UNVA of 20/30. Additionally, 84% attained simultaneous UDVA of 20/25 and UNVA of J1. A loss of one line of CDVA occurred in 9.45% of eyes, while 4.05% lost two or three lines at the 6-month examination. Postoperative optical changes included increased negative spherical aberration and vertical coma. Overall patient satisfaction was high, with 83.3% reporting that they were pleased with the visual outcome [131].

Cosar et al. presented 6-month results showing that 77.2% of eyes achieved an UNVA of 20/20 and 89.4% achieved 20/25. However, 28.5% of eyes lost one line of CDVA and 10.6% lost two lines [132].

- PresbyMAX SCHWIND

PresbyMAX is a modern presbyLASIK platform based on a bi-aspheric multifocal corneal profile designed to extend depth of focus while maintaining high-quality distance and near vision. Its optical architecture incorporates a central hyperpositive zone (+0.75 to +2.5 D) dedicated to near focus, surrounded by a smooth peripheral transition optimized for distance vision. Unlike earlier multifocal LASIK concepts, which relied on abrupt changes in corneal power, PresbyMAX utilizes gradual power transitions, thereby reducing induced spherical aberration and minimizing degradation of optical quality [125,133,134].

The platform offers three algorithmic configurations tailored to binocular visual strategy and patient-specific needs.

PresbyMAX Symmetric applies an identical multifocal profile to both eyes, supporting binocular summation and preserving stereopsis [125,135].

PresbyMAX Hybrid enhances distance optimization in the dominant eye while providing greater near addition in the nondominant eye.

PresbyMAX µ-Monovision combines mild monovision with multifocality, offering extended depth of focus with minimal compromise in binocular distance acuity [133,134].

These customizable modes allow the clinician to balance near and distance visual demands across diverse refractive categories.

Uthoff et al. evaluated the PresbyMAX technique in hyperopic, myopic and emmetropic presbyopes. At 6 months, 83% of all patients achieved a UDVA of 0.1 logMAR or better, including 100% of hyperopic, 80% of emmetropic and 70% of myopic patients. Uncorrected near acuity of 0.3 logRAD or better was reached by 90% of emmetropic eyes and by 80% of both hyperopic and myopic eyes. Loss of best-corrected distance visual acuity (BCDVA) varied by refractive group: in the hyperopic and emmetropic cohorts, 10% of patients lost two lines and 40% lost one line, whereas in the myopic cohort, 10% lost three lines, 10% lost two lines, and 10% lost one line. A shift toward increased negative spherical aberration was observed, although third-order coma and trefoil remained unchanged. The hyperopic group reported the highest satisfaction. No retreatments were required within the 6-month observation period [136].

Luger et al. later reported 1-year outcomes of PresbyMAX in myopic and hyperopic patients, with or without astigmatism. At one year, 70% of patients achieved a UDVA of 0.1 logMAR or better, and 84% achieved an UNVA of 0.1 logRAD or better. Additionally, 85% reached both UDVA of 0.2 logMAR and UNVA of 0.2 logRAD or better. Loss of two lines occurred in 3% of eyes for CDVA and in 8% for corrected near visual acuity (CNVA) [137].

Baudu et al. analyzed binocular outcomes 6 months after PresbyMAX in hyperopic and myopic presbyopic patients. They reported that 76% of patients achieved a binocular UDVA of 0.1 logMAR or better, while 91% achieved an UNVA of 0.1 logRAD or better. Binocular success—defined as both UDVA ≤ 0.15 logMAR and UNVA ≤ 0.15 logRAD—was attained in 80% of cases [138].

In a separate investigation, Luger et al. evaluated PresbyMAX combined with micro-monovision in both myopic and hyperopic presbyopes over a 1-year follow-up. Target refraction was −0.1 D in the dominant (distance) eye and −0.9 D in the non-dominant (near) eye. Ninety-three percent of patients achieved a UDVA of 20/20, 90% achieved a UNVA of J2, and 97% attained an uncorrected intermediate visual acuity (UIVA) of J2. A loss of two Snellen lines of CDVA was observed in 7% of patients [137].

Chan et al. reported 1-year outcomes of a hybrid approach combining PresbyMAX in the non-dominant eye with monofocal distance correction in the dominant eye for bilateral hyperopic presbyopes. At one year, 87% of patients achieved a UDVA of 20/25 or better and 83% reached an UNVA of J2 or better. Binocular simultaneous distance and near vision (20/25 and J2 or better) was obtained in 70% of patients. No eye lost two lines of CDVA, although 14% required retreatment for near vision enhancement within the first postoperative year. The procedure induced a significant increase in negative spherical aberration, and the change in total HOA was significantly different between fellow eyes. Overall satisfaction was high, with 94% of patients reporting satisfaction, although 26% noted reduced visual performance under low-illumination conditions [139].

Across multiple prospective and retrospective studies, PresbyMAX demonstrates consistently favorable binocular visual outcomes. Intermediate vision (UIVA) is also robust due to effective extension of depth of focus, making PresbyMAX suitable for daily visual tasks that rely on functional mid-range acuity. Importantly, favorable outcomes are consistently observed even in hyperopic presbyopes, a population historically challenging to treat with refractive solutions.

Retreatment rates following PresbyMAX generally range between 10% and 19% within the first postoperative year. Most enhancements address residual refractive error rather than intolerance to multifocality and true reversals due to neural adaptation issues are relatively uncommon at approximately 3%. Higher enhancement rates are noted in preoperative hyperopes, consistent with known refractive instability patterns in this subgroup [125,133,134,136].

Refractive stability with PresbyMAX is well documented. Studies with 12- to 36-month follow-up consistently show stable refraction without significant regression [134,135]. Long-term data extending to five years confirm sustained distance and near acuity, with only mild hyperopic drift observed in initially hyperopic patients. This trend reflects general LASIK biomechanical behavior rather than limitations of the PresbyMAX algorithms themselves [135].

Quality-of-vision outcomes are similarly encouraging. Photopic and mesopic contrast sensitivity remains largely preserved, with only mild scotopic reductions noted in some individuals [134,140]. Subjective visual disturbances such as halos and glare tend to be most prominent in the early postoperative period but typically diminish by three to six months, attributable to neural adaptation processes rather than persistent optical imperfections [125,133,134,139]. Compared with earlier generations of presbyLASIK designs, PresbyMAX exhibits reduced night-vision symptoms and less pronounced contrast sensitivity loss, owing to its smoother and more progressive optical transitions [140].

Collectively, the current body of evidence indicates that PresbyMAX provides predictable binocular vision restoration, durable refractive stability and a relatively favorable quality-of-vision profile. Its customizable multifocal algorithms offer surgeons a flexible and effective approach to addressing presbyopia across a broad spectrum of refractive presentations.

- Presbyond

The Presbyond technique integrates non-linear aspheric corneal ablation, deliberate induction of spherical aberrations in both eyes and micro-monovision of −0.75 to −1.5 D in the non-dominant eye [126,140,141].

This procedure was introduced by Reinstein et al. [142,143,144].

It has proven effectiveness and safety in the correction of presbyopia, regardless of the preoperative refractive state and is EMA (European Medicines Agency)-approved for adults older than 40 years presenting with myopia (≤−8.0 D), emmetropia, hyperopia (≤+4.0 D), astigmatism (≤2.5 D), and a positive results on the cross-blur monovision tolerance test [119,142,143,144].

Thanks to the combination of an aspheric ablation profile based on preoperative spherical aberration patterns and micromonovision, by increasing depth of field, Presbyond delivers excellent visual acuity across near, intermediate, and distance ranges [119,140].

Compared to traditional monovision, micromonovision involves limiting the near power in the non-dominant eye to no more than +1.5 Dsph, regardless of the individual’s age [142,143,144].

The controlled induction of spherical aberrations allows for the creation of an optimized ablation profile based on factors such as age, preoperative refractive state, tolerance to anisometropia, preoperative spherical aberration and centration [119,140].

According to experimental studies by Rocha et al., carried out using adaptive optics simulators, a controlled bilateral increase in spherical aberration up to <0.56 µm resulted in an extension of the corneal refractive power depth by about 1.5 D, with the brain potentially able to filter blurred images following neuroadaptation [145].

Furthermore, the introduction of spherical aberration enhances visual clarity in the eye corrected for near vision, when defocus is present.

As a result, the non-dominant eye achieves superior intermediate and distance vision compared to what is observed in traditional monovision [119,140].

Additional factors also play a role in the high effectiveness and strong safety profile of this refractive procedure. These mechanisms involve the constriction of the pupil during accommodation, changes in the corneal epithelium, the variation in refractive indices between the corneal epithelium and stroma and the binocular integration in the visual cortex that develops over several months of neuroadaptation [119,140].

All aforementioned, result in the formation of a blend zone, that ensures continuous good binocular vision for near, intermediate and far distances [119,140].

The Presbyond procedure is performed using the femtoLASIK technique and the MEL 90 or MEL 80 excimer laser systems, along with the CRS Master platform [142,143,144].

Proper patient qualification is key to achieving satisfactory results [142,143,144]. The criteria for selection include:

- normal corneal topography, with no signs of ectasia,
- corrected distance visual acuity (CDVA) of at least 20/25 in both eyes,
- an anisometropia tolerance minimum 0.75 Dsph,
- effective binocular integration and suppression,
- strong motivation with realistic expectations.

Contraindications for the procedure include:

- corneal haze and lens opacities,
- severe dry eye syndrome,
- pathologies of the optic nerve or retina,
- acute/chronic systemic diseases,
- diseases involving immunosuppression.

Before the procedure, it is essential to assess eye dominance and tolerance for micromonovision within the range of 0.75 Dsph to 2.0 Dsph, usually around 1.5 Dsph [142,143,144].

The results of the Presbyond procedure, as presented by Reinstein et al., show that in a 1-year follow-up, the best visual acuities were achieved by patients with preoperative myopia ranging from −0.2 to −8.3 Dsph [144].

In this group:

- 99% achieved binocular uncorrected distance vision of 20/20 and uncorrected near vision of J5,
- none of the patients experienced a loss of more than 2 lines of CDVA,
- postoperative refractive shifts between 3 months and 1 year ranged from −0.06 ± 0.31 Dsph [144].

Comparable results were observed in emmetropic individuals, where

- 98% reached 20/20 binocular UDVA,
- 96% achieved near vision of J2 or higher without correction,
- none of the patients experienced a decrease of over 2 lines in preoperative CDVA [143].

In patients with preoperative hyperopia, results were slightly worse:

- 90% achieved uncorrected distance vision of 20/20 and uncorrected near vision of J5,
- none of the patients experienced a decrease of over 2 lines in CDVA,
- postoperative refractive shifts between 3 months and 1 year ranged from +0.11 to ±0.36 Dsph [142].

Ganesh and Brar studied patient satisfaction with Presbyond outcomes 1 year post-procedure. They reported: 97% of patients with myopia and 96% of those with hyperopia were satisfied with UDVA, 95% of patients with myopia and 89% of those with hyperopia were satisfied with UNVA [146].

According to analysis conducted by Wierzbowska et al., Presbyond does not impair contrast sensitivity in any of the treated groups of presbyopic patients—myopic, emmetropic, or hyperopic [119,140].

Surgically induced anisometropia can lead to suppression, impaired fusion and loss of stereopsis, which is observed after procedures resulting in conventional monovision [140].

During the Presbyond procedure, a personalized ablation profile and optimized levels of anisometropia, as well as spherical aberrations, protect against loss of retinal correspondence, enabling neural binocular summation, and thus preserving functional stereopsis [119,140].

The impact of the Presbyond procedure on stereoscopic vision has not been definitively established in the studies conducted so far, whose results are limited and varied [140,147].

After Presbyond treatment, stereopsis can be enhanced by refractive errors correction, reducing anisometropia and eliminating unwanted effects of glasses such as minification, magnification, and prismatic distortion [140,148,149,150,151].

In an analysis performed by Wierzbowska et al., both—cases of significant postoperative deterioration and improvement in stereoacuity scores—were observed [140,147]. The analysis included existing research on the influence of the Presbyond procedure on stereoscopic vision. The authors summarized the findings by categorizing the reviewed studies into two groups based on follow-up duration: 3 months and 6 months or longer. In the 3-month follow-up group, two studies reported a significant decline in distance stereopsis, while one of these studies also observed a significant improvement in near stereopsis. In the studies with a follow-up period of 6 months or longer, postoperative stereopsis remained unchanged in one study and decreased in two others [140]. The authors emphasized that the Presbyond LBV procedure may be associated with a temporary reduction in stereopsis, but it ensures preserved stereoscopic function in the majority of patients [140,147].

According to Zhang et al., near stereopsis showed an unspecified improvement three months postoperatively. Meanwhile a statistically significant postoperative reduction in stereopsis to distance was perceived [152].

Romero et al. found that stereopsis significantly improved six months following Presbyond treatment, but this effect was limited to eyes with moderate myopia (−3.0 to −6.0 D) [153]. In eyes with hyperopia or low myopia, stereoscopic vision remained unchanged [153].

A significant reduction in stereopsis was observed postoperatively by Russo et al., but still remaining within the functional range [154]. According to these authors 79% of myopic patients and 85% of hyperopic patients retained functional stereoscopic vision (stronger than 100 arcseconds) six months after surgery [154].

According to Reinstein and colleagues, even though uncorrected stereoacuity after surgery was lower than near-corrected stereoacuity before the procedure, 68% of patients 6 months after Presbyond had a stereoacuity of 100 arcseconds or better [155].

According to Brar et al. stereoacuity of 70% patients six months after Presbyond was not less than 60 arc of seconds, however the authors observed a notable reduction in uncorrected stereopsis, which reached preoperative levels when assessed with the near addition [156]. These authors also found that reading speeds six months after Presbyond procedure is better at every distance. Moreover according to aforementioned authors, mean satisfaction indices were 98.0 ± 2.1 for distance, 99.46 ± 0.6 for intermediate, and 96.8 ± 2.4 for near vision. Satisfaction rates among patients were 93.3% for distance, 100% for intermediate, and 86.6% for near vision [156]. All patients reported the absence of glare or halo effects [156]. At the end of the 6-month follow-up, 2 patients (6.6%) reported mild dysphotopsia (grade 1) [156].

Short-term side effects of the Presbyond technique include: increased dryness of the eyes, glare, visual acuity fluctuations within 3–4 months post-surgery [126].

Current observations confirm the durability of postoperative effects, safety and good tolerance of the procedure for up to 12 months in presbyopic patients, regardless of their preoperative refractive state, with no significant adverse effects [142,143,144,145,146].

Table 2 summarizes the key characteristics and clinical outcomes of two laser presbyopic correction approaches—PresbyMAX and Presbyond.

- INTRACOR

INTRACOR utilizes a femtosecond laser to treat presbyopia in individuals with emmetropia.

Ruiz and colleagues first described this technique [157].

During INTRACOR, pulses from a femtosecond laser are delivered in a concentric pattern into the corneal stroma, performing 5 circular incisions around the visual axis and resulting in a change in its shape without the need for creating a flap or disrupting the continuity of the epithelium [157].

The procedure’s minimal invasiveness, lack of pain and rapid recovery are significant advantages of the INTRACOR technique [157].

Over a 36-month period, Thomas et al. followed 25 presbyopic patients who received the INTRACOR procedure in the non-dominant eye [158].

This analysis showed an improvement in UNVA from a preoperative value of 0.7 logMAR to 0.1 logMAR at 36 months postoperatively, accompanied by a loss of 1 line in UDVA [158].

Additionally, a progressive myopic shift was observed, amounting to 0.5 D at 36 months after the procedure, as well as significant corneal steepening in the treated area, reaching 1.5 D [158].

Corneal thickness increased from 535 µm before surgery to 549 µm at the final follow-up. This increase was considered statistically significant but clinically insignificant [158].

No changes in endothelial cell density were observed [158].

92.86% of the patients reported comfort related to the procedure [158].

Despite its effectiveness and minimal invasiveness, INTRACOR is not free from side effects.

Some of the disadvantages include: reduced contrast sensitivity under mesopic conditions, increased glare, potential negative effects on driving ability in low-light conditions [159,160].

Moreover, possible combinations of INTRACOR with other laser refractive surgery techniques may induce ectasia in eyes that do not have pre-existing risk factors for its development [161]. INTRACOR currently does not have FDA approval as a method for presbyopia correction [162].

- CustomQ Wavelight Refractive Suite Platform (Alcon Laboratories Inc., Geneva, Switzerland)

Custom-Q is a corneal refractive technique in which negative corneal asphericity is induced in the non-dominant eye to enhance near vision. The procedure uses a nomogram to select the target refraction and degree of negative spherical aberration, allowing individualized treatment planning. Clinical outcomes in hyperopic and presbyopic patients have shown promising results: for example, in a series of 65 hyperopic presbyopes treated with a custom-aspheric ablation profile and micro-monovision, 91% of patients achieved binocular uncorrected distance visual acuity (UDVA) of 20/20 or better and 83% achieved binocular uncorrected near visual acuity (UNVA) of J3 (or better) at 6-month follow-up [163].

Another independent series on presbyopic patients reported mean binocular UDVA around 0.15–0.11 logMAR (≈20/25) and binocular UNVA of about 0.5–0.45 M (≈J2) at 6 months, with stereopsis improved postoperatively [164].

#### 4.3.2. Refractive Lens Exchange (RLE) in Presbyopia Correction

Refractive lens exchange (RLE) has evolved beyond cataract removal, now serving as an effective method to correct refractive errors and alleviate presbyopia symptoms [165]. Advances in surgical safety, outcomes and intraocular lens (IOL) technology have made RLE increasingly popular for presbyopia correction [166].

Currently, approximately 85% of RLE patients achieve postoperative refractive results within ±0.5 D of the target, compared to only 60% in the early 2000s [165,167]. Modern IOL designs have shifted both patient expectations and surgical criteria, aiming for spectacle independence and improved visual quality [166].

Advantages of RLE include:

- Independence from spectacles, either partially or fully [166],
- Effective correction of high refractive errors unsuitable for corneal refractive surgery [166],
- Prevention of future cataract development [166].

Proper patient selection is critical for success. Preoperative evaluation should include a comprehensive assessment of visual goals, daily activities, occupational requirements and personality traits [166,168]. Tools such as SimVis Gekko or VirtIOL visual simulators may aid in counseling patients regarding expected outcomes, though further clinical validation is needed [169,170]. A thorough preoperative examination must evaluate the cornea, iris, pupil, lens, ciliary apparatus, retina and optic nerve, as ocular comorbidities may influence postoperative outcomes [168].

Ideal candidates are typically patients over 50 years, with moderate hyperopia (+3 to +4 D) and early lens changes, who benefit from both presbyopia correction, and early cataract management [166,168]. In younger high-myopia patients, RLE carries higher risks, including retinal detachment and may yield lower satisfaction due to adequate preoperative near vision or altered depth perception [168].

RLE can utilize a variety of IOLs tailored to individual visual requirements.

This IOLs proliferation, while benefiting patient care, necessitated a clear, standardized classification system.

Historically, IOLs were often categorized by their optical design, a method that frequently lacked direct correlation with the patient’s functional visual outcomes in the real world.

To address this, the European Society of Cataract and Refractive Surgeons (ESCRS) Functional Vision Working Group developed an evidence-based, functional IOL classification [171]. This system shifts the focus from the lens mechanism to the achieved Range of Field (RoF), using objective metrics derived primarily from defocus curves.

The ESCRS classification is scientifically rigorous, grounded in a comprehensive scoping review and cluster analysis of published defocus curve data. This data-driven approach identified two core metrics essential for classification:

- Range of Field (RoF): The continuous dioptric range over which a predefined level of high visual acuity (typically ≤0.2 logMAR or ≤0.3 logMAR) is maintained.
- Visual Acuity Difference (ΔVA): The difference in visual acuity between intermediate and near distances for lenses aiming at spectacle independence (Full RoF), used to characterize the transition smoothness

The system divides all IOLs into two primary functional categories, with subcategories that directly correlate with both the RoF and the commonly used industry nomenclature:

Partial Range of Field (Partial RoF) IOLs

These lenses provide excellent distance vision and a variable, but non-full, range of vision into the intermediate zone. Patients typically require spectacle correction for near vision. Among them are:

- Narrow RoF: Corresponds to Standard Monofocal IOLs, offering a single, sharp focal point with minimal extension.
- Enhanced RoF: Corresponds to Monofocal-plus (Monofocal+) IOLs (sometimes referred to as low-add EDOF). These provide a measurable extension of focus into the intermediate distance (e.g., 66 cm), improving functional vision without compromising distance acuity.
- Extended RoF: Corresponds to EDOF (Extended Depth of Focus) IOLs and Hybrid-EDOF designs. These lenses provide a broader, continuous range of sharp vision from distance into the intermediate zone (often approaching 40 cm), characterized by the absence of a distinct peak for near vision.

Full Range of Field (Full RoF) IOLs

These lenses are designed to deliver vision across the entire functional range—distance, intermediate and near, with the primary goal of achieving high levels of spectacle independence. These are often referred to as simultaneous vision lenses (SVLs). Subclassification here depends on the nature of the transition between the focal points:

- Continuous: Lenses offering a smooth, minimal variation in visual acuity across all distances, indicating a highly integrated focus.
- Smooth Transition: Lenses, typically trifocal and certain multifocal designs, where the difference between intermediate and near foci is moderate.
- Steep Transition: Lenses, often traditional bifocal designs, which exhibit a pronounced, sharp transition between distinct focal points (distance and near).

The ESCRS functional classification provides a unified, evidence-based language that bridges the gap between technological design and clinical outcome. This modern functional approach is positioned to become the global standard for classifying IOL performance, ultimately enhancing both surgical decision-making and patient satisfaction.

A comprehensive 2024 network meta-analysis of randomized controlled trials (RCTs) compared monofocal, enhanced monofocal, EDOF and multifocal IOLs in patients undergoing cataract surgery or refractive lens exchange (RLE) [172]. The analysis demonstrated that trifocal IOLs provide superior uncorrected near visual acuity (UNVA) compared with monofocal lenses, with a mean difference of approximately −0.35 logMAR [173]. Both EDOF and trifocal IOLs yielded significantly better uncorrected intermediate visual acuity (UIVA) than monofocal IOLs, including models such as AcrySof PanOptix trifocal and contemporary EDOF lenses [172]. Spectacle independence rates were highest with trifocal IOLs, whereas EDOF and enhanced monofocal IOLs offered a favorable balance between intermediate vision and overall optical quality, although near acuity was sometimes slightly lower than with multifocals [172,173,174].

To date, the only enhanced-monofocal intraocular lens with published long-term (5-year) follow-up data is Tecnis Eyhance. In a cohort of 36 eyes (18 patients) the 5-year data showed stable uncorrected distance, intermediate and near visual acuity, preserved contrast sensitivity, and optical quality, consistent effective lens position, low posterior-capsule opacification rate (5%), mild halos/glare, and high spectacle independence for distance and intermediate vision [175].

A recent retrospective study comparing a modern purely refractive EDOF IOL (PureSee/ZEN00V) with an enhanced monofocal IOL (Eyhance/ICB00) found superior uncorrected intermediate and near acuity in the EDOF group, while distance acuity and contrast sensitivity were comparable [176]. Spectacle dependence for near vision was markedly lower in the EDOF group (36% vs. 80%), although a slightly higher incidence of photic phenomena, such as halos and glare, was reported, remaining within acceptable limits. A broad systematic review of presbyopia-correcting IOLs (monofocal plus, EDOF, multifocal) reinforced these findings: trifocal IOLs deliver the best near vision but are associated with increased risk of photic phenomena and reduced contrast sensitivity, whereas EDOF and enhanced monofocal IOLs provide improved intermediate vision, fewer dysphotopsias and superior overall optical quality [177].

Based on current evidence and the ESCRS classification, patients seeking excellent distance and intermediate vision with minimal photic phenomena and good contrast are well suited for enhanced monofocal (monofocal plus) or EDOF IOLs. Those prioritizing spectacle independence for near vision, such as reading or close work and willing to accept potential dysphotopsias may benefit most from full RoF IOLs. In contrast, patients with ocular surface disease, high-risk retina (e.g., myopia), or limited tolerance for optical trade-offs should generally receive standard monofocal IOLs. Comprehensive preoperative counseling is essential to ensure patients understand the balance between visual range, optical quality and the risk of photic phenomena [177].

The main causes of dissatisfaction with the outcomes of RLE using F-RoF and EDOF—so called “premium” lenses are: quality of vision, photic phenomena under mesopic conditions and loss of contrast sensitivity.

Characteristic photic phenomena associated with premium lenses are positive dysphotopsias, which are bright artifacts around light sources. These include halos and glare.

According to published data, the occurrence of halos and other photic phenomena demonstrates considerable variability, spanning from below 1% to 89% [178,179,180,181,182].

In the study by Garcia-Perez and coworkers, most patients (84.5%) experienced no vision problems after RLE with multifocal lenses, although 32.8% reported halos in mesopic conditions and 10.3% experienced glare [178]. In contrast, Kohnen observed that photic phenomena occurred in 93% of patients, comprising 89% halos around light sources, 11% glare, 7% diplopia and 4% visual shadows or distortions [183].

Rosen et al. report that photic phenomena occur more frequently with trifocal lenses than with bifocal lenses and that patients tolerate them significantly better six months after implantation [184].

According to Lawless and colleagues, moderate halos were present in 15% of patients post-procedure, without impacting their daily functioning [185]. This phenomenon decreased within 2–3 months post-surgery [185].

The occurrence of photic phenomena is almost inevitable in patients receiving multifocal intraocular lenses. However, the majority are experienced as non-problematic and their perception typically decreases with time [168,185,186].

According to Kretz et al., one month after trifocal lens implantation, halos were reported by 90% of patients, although 80% of them described these halos as not interfering with daily life [187].

Three months after the procedure, this phenomenon was observed in only 50% of patients [187].

Furthermore, the reduction in halo effect is also attributed to neuroadaptation [168,188].

Neuroadaptation occurs due to the plasticity of the cerebral cortex, which allows suppression of blurred images arising from the additional second and/or third focal points of the lens [168,188].

According to functional magnetic resonance (fMRI) studies, immediately after implantation of multifocal lenses, brain regions responsible for learning, attention and solving complex tasks—the cingulate gyrus, frontal and parietal lobes—exhibit increased activity, which normalizes after approximately six months [189]. Afterwards, the perception of dysphotopsia usually decreases [168].

A common drawback of multifocal lenses is that light from out-of-focus images can reduce the contrast of the in-focus image. The source of contrast reduction is the uneven distribution of light [188,190].

Research indicates that multifocal lenses are associated with lower contrast sensitivity for high spatial frequencies in dim lighting conditions compared to monofocal lenses [168,190].

However, to date, no significant impact of this reduction in contrast sensitivity on patients’ daily functioning, has been observed [168,184].

Patients’ age can influence satisfaction with the outcome of RLE surgery.

Younger patients with presbyopia usually lead more active lifestyles and demand good visual acuity at all distances—near, intermediate and far. Older presbyopic individuals, experienced in using glasses for near tasks over many years, might be more accepting of slight compromises in visual clarity to achieve improved near vision. However, elderly patients may experience a delayed vision restoration following surgery and a greater risk of postoperative adverse events [166,168].

Schallhorn et al. in their analysis of RLE outcomes, categorized patients into four age brackets: 45–49, 50–54, 55–59 and 60–65 years. The proportion of patients achieving binocular UDVA of 20/20 or better was 91.6%, 93.8%, 91.6% and 88.8%, respectively. Binocular UNVA of 20/30 or better was observed in 80.0%, 84.7%, 78.9%, and 77.8% of patients across the respective age groups [191].

The authors reported that the severity of photic phenomena and difficulties with night driving did not differ significantly between age groups [191].

The highest rate of lens explantation due to dissatisfaction with postoperative visual quality was observed in age group A, at 1.3% level. In the other age groups, this rate was up to 0.6% [191].

Postoperative complications observed after RLE procedures include:

- residual refractive error,
- incorrect positioning of the intraocular lens,
- posterior capsular opacification,
- retinal detachment,
- cystoid macular edema,
- endophthalmitis [166,168,192].

Residual refractive error

Residual astigmatism after multifocal intraocular lens implantation is a leading factor contributing to patient dissatisfaction [192].

The detrimental effect of uncorrected corneal astigmatism on visual outcomes after monofocal intraocular lens implantation has been well documented in prior studies. This relationship is even more pronounced for multifocal lenses. In the case of bifocal and trifocal lenses, visual acuity is particularly affected by astigmatism of ≥1.0 D and ≥0.75 D, respectively [193,194,195,196]. Therefore, to optimize visual outcomes, patients with predicted postoperative astigmatism greater than 1.5 D for with-the-rule and 0.75 D for against-the-rule, should undergo an astigmatism-correcting intervention at the time of multifocal lens implantation. Options include a clear corneal incision at the steepest meridian, an opposed incision, or implantation of a multifocal toric intraocular lens [197].

Meta-analytic evidence from randomized trials shows, that toric lenses achieve better UDVA, reduced residual astigmatism and higher independence from spectacles than combination of non-toric lenses with limbal relaxing incisions [198].

Postoperative refractive outcome is one of the key factors determining satisfaction with vision after surgery. Its value is influenced by [168]:

- Proper preoperative preparation, including corneal tomography to assess total corneal astigmatism, as well as biometry and calculation of the implanted lens power,
- Surgical technique:
  - The characteristics of the corneal incision, including its location, length, and width, are key factors influencing surgically induced astigmatism (SIA) [199],
  - The final positioning of the IOL depending on anterior capsulorhexis dimensions and configuration.

Incorrect positioning of the intraocular lens

The dimensions and positioning of the anterior capsulorhexis, markedly affect lens stress, tilt and decentration [200,201].

Findings from Cornaggia and coworkers indicate that greater capsulorhexis width and decentration, result in increased lens tilt [200].

Rossi et al. found that deviations in capsulorhexis regularity and alignment lead to lens tilt and decentration, spanning roughly 10^−1^ to 10^−7^ degrees and 10^−2^ to 10^−7^ mm [201].

When postoperative refractive outcomes deviate from the intended target, potentially reducing patient satisfaction, corrective interventions such as corneal laser refractive surgery, lens exchange, or piggy-back lens implantation may be considered [168].

Posterior capsule opacification

A complication that significantly reduces patient satisfaction with the outcome of RLE is posterior capsule opacification (PCO) [192].

Lens parameters such as material, edge design and haptic construction determine the risk of developing PCO [192,202,203,204].

Protective measures to prevent PCO include thorough cortex cleaning, covering the optical part of the IOL with a 1 mm rim of the lens capsule (capsulorhexis edge) over 360 degrees and implanting a hydrophobic lens with square edges [202,203,204]. To remove PCO, laser posterior capsulotomy is performed [192,202,203,204].

In a retrospective analysis conducted by Stonecipher et al., posterior capsulotomy was performed in 36% of eyes undergoing RLE [202]. A significant variation in PCO incidence was observed depending on the preoperative refractive error. Posterior capsulotomy was most frequently performed in patients with preoperative myopia (42%), followed by those with astigmatism or emmetropia (40%) and least often in patients with hyperopia (16%) [202].

Shah and colleagues observed that multifocal lens implantation was associated with a significantly higher posterior capsulotomy rate compared to monofocal lens implantation [203]. Over the observation period of 2–41 months 15.49% of patients in the multifocal lens group and 5.82% in the monofocal lens group underwent posterior capsulotomy [203].

This difference is due to the negative impact even a slight opacification of the posterior capsule has on visual quality in eyes with implanted multifocal lenses [204].

Retinal detachment

Retinal detachment is a complication potentially threatening vision.

Based on existing evidence, the cumulative 10-year risk of retinal detachment following uneventful phacoemulsification, ranges between 0.36% and 2.9%. Despite a gradual decline to 0.1–0.2% annually, the rate still exceeds that of the general population by approximately tenfold [205].

A higher incidence of retinal detachment following phacoemulsification has been linked to certain predisposing factors: intraoperative vitreous loss, elongated axial length of the eyeball, younger patient age, male sex and surgeon experience [205].

Among highly myopic eyes undergoing phacoemulsification, the 2-year likelihood of retinal detachment ranges from 1.5% to 8.1%, with a higher rate observed in patients under 40 years of age [205]. The association between retinal detachment after RLE in highly myopic patients may result from preexisting peripheral retinal pathologies—degenerations, holes, tears—which are more common in myopic eyes, as well as intraoperative volumetric changes in the vitreous body [206].

In young highly myopic individuals without posterior vitreous detachment (PVD), the occurrence of retinal detachment after RLE is increased [205].

According to Alio and colleagues, retinal detachment after RLE was more common in younger patients (≤50 years) and in highly myopic eyes with axial lengths exceeding 28 mm. The study included patients grouped by age (≤50 and >50 years) and axial length (≤28 mm and >28 mm) [207]. In this study, after RLE, retinal detachment was more frequent in patients aged 50 years or younger (3.65%) compared to those over 50 years (2.52%) [207].

Cystoid macular edema

Cystoid macular edema (CME), according to reports by Schallhorn et al., occurs after RLE with an incidence of 0.29% [208].

It is a complication, that can significantly negatively impact postoperative satisfaction outcomes following RLE.

The occurence of cystoid macular edema after phacoemulsification cataract surgery is associated with several risk factors, including older age, male sex, diabetes, uveitis, epiretinal membrane, a prior history of retinal detachment and surgical complications [208].

Endophthalmitis

Endophthalmitis is considered the most severe complication associated with eye surgery. Intraoperative intracameral antibiotics have significantly reduced the incidence of endophthalmitis after phacoemulsification [209].

According to study by Schallhorn and coworkers, only one case of postoperative endophthalmitis after RLE was reported, in which visual acuity permanently decreased to 20/50 [208].

This very low incidence, much lower than that seen after cataract removal surgery (0.04–0.2%), is likely due to the younger age and good overall health of the operated population, as well as the intraoperative intracameral administration of cefuroxime and the low rate of intraoperative complications [208].

##### Refractive Lens Exchange in Presbyopia Correction Using a Light-Adjustable Intraocular Lens

In refractive lens exchange (RLE), the use of an intraocular lens (IOL) with postoperatively adjustable power represents a major advancement in the individualized correction of ametropia. The Light Adjustable Lens (LAL) remains the only IOL capable of modifying the refractive target after implantation. The newest generation of this platform, including the LAL+ variant, employs a photosensitive polymer optic that can be reshaped with ultraviolet (UV) light to achieve the desired postoperative refraction. The LAL+ introduces a modest, continuous increase in central optical power relative to the standard LAL, resulting in a broadened depth of focus (DoF) while maintaining the same high-quality distance vision demonstrated in optical simulations. Since its release in late 2023, the LAL+ has offered surgeons and patients an expanded range of vision without compromising the adjustability inherent to the LAL technology [210].

Adjustment is typically performed after a 2–3-week postoperative healing period, during which the cornea stabilizes. UV irradiation then induces polymerization within the lens, leading to precise changes in lens shape and power according to the planned refractive target. When optimal vision is achieved, a final “lock-in” exposure permanently fixes the adjusted configuration and prevents further changes from ambient UV light [210].

Recent multicenter data highlight the high refractive predictability and functional performance of both LAL and LAL+. Approximately 91.1% and 93.5% of LAL, and LAL+ eyes, respectively, achieve a manifest spherical equivalent within ±0.50 D of target, and roughly 92% of patients reach binocular uncorrected distance visual acuity (UDVA) of 20/20 or better after adjustment. Best-focus binocular near acuity of J1 or better at high contrast is observed in 86–93% of cases, with LAL+ showing further enhancement of intermediate vision owing to its expanded DoF [210].

Similarly, in eyes with prior corneal refractive surgery—a historically challenging population for IOL power prediction—the LAL demonstrates strong performance: one 2024 series of 94 eyes reported UDVA of ≥20/25 in 82% and 20/20 in 74% of eyes, with 88% and 69% of cases reaching the target spherical equivalent within ±0.50 D and ±0.25 D, respectively [211].

Additional support for the performance and safety of the LAL comes from a Japanese retrospective study evaluating 34 eyes from 21 patients, including eyes with and without prior refractive surgery. The mean prediction error following adjustment was −0.04 ± 0.48 D. Spherical equivalents within ±0.25 D and ±0.50 D were achieved in 91% and 97% of eyes, respectively, and 94% exhibited ≤0.50 D of residual astigmatism. A functional DoF of 3.68 D was recorded, and contrast sensitivity remained comparable to that of an age-matched normal population. Patient satisfaction was high (mean 8.8/10) and no adverse events or losses of corrected distance acuity were observed [212].

Long-term safety and refractive stability have been assessed in a noninterventional observational study conducted at the University Eye Hospital in Bochum, Germany. Among 445 patients implanted with the LAL from 2008 to 2012, 61 patients (103 eyes) participated in follow-up examinations an average of 7.2 years after lock-in. Both corrected and uncorrected distance acuity remained good, and refractive outcomes were stable with minimal deviation from the final adjusted values. Corneal thickness showed no significant changes and only two patients demonstrated mild IOL opacities without impact on visual acuity. Other ocular findings were consistent with age-related expectations, confirming long-term structural and functional safety [213].

Taken together, the accumulated evidence demonstrates that the LAL and LAL+ platforms provide uniquely precise, customizable refractive outcomes with excellent visual performance across distances, high patient satisfaction, and reassuring long-term safety. The consistency of results across diverse patient groups—including post-refractive surgery eyes, and across follow-up periods exceeding seven years, underscores the robust and individualized nature of this technology.

While the LAL offers clear advantages over fixed-power IOLs, its use requires patient adherence to wearing UV-blocking eyewear until lock-in, as well as awareness that optimal vision emerges only after the adjustment process is completed. Although long-term real-world data are steadily expanding, they remain more limited compared with established monofocal and multifocal technologies. Nonetheless, modern LAL technology stands out as the only IOL system capable of achieving postoperative refractive fine-tuning, delivering high accuracy, excellent distance and intermediate acuity, and strong overall visual quality—positioning it as a transformative option in refractive cataract surgery and RLE.

## 5. Dynamic Methods in Presbyopia Correction

Dynamic techniques utilize the potential of ocular structures involved in the accommodation process to maintain the eye’s capacity for real-time adjustment of optical power according to the distance to the object being observed [33].

Evidence indicates that in presbyopia, the ciliary muscle preserves its contractile capacity and the lens envelope remains flexible, even with advancing age. These structures serve as key targets for dynamic presbyopia correction techniques [214,215,216].

Procedures using a dynamic approach to presbyopia correction include scleral surgery techniques and RLE with implantation of accommodating intraocular lenses.

### 5.1. Scleral Surgery in Presbyopia Correction

Scleral surgery enables the restoration of the physiologically natural accommodation process [33,217].

Historically, the foundations of scleral surgery used for presbyopia correction were based on Schachar’s model of accommodation.

Procedures such as anterior ciliary sclerotomy (ACS) were designed to widen the distance between the ciliary muscle and lens equator, in order to restore the eye’s age-related loss of accommodative amplitude [26,218,219,220,221,222].

However, the effects of these procedures were short-lived, which prompted further research aimed at modifications to prevent rapid regression [218].

One such modification was introduced by Fukasaku, who used silicone implants inserted into the scleral incision sites [223].

Currently, these procedures are no longer used for presbyopia correction [224].

Another attempt to compensate for presbyopia through scleral surgery, also inspired by Schachar’s model, were scleral expansion bands (SEBs) [222,225].

The implants were intended to expand the ciliary-scleral interface, with the goal of restoring the eye’s diminished accommodative range [222,225].

During the procedure, rods made of polymethyl methacrylate (PMMA) were used [222,225].

A complication of SEB was episodes of anterior segment ischemia, which temporarily led to a nearly complete decline in interest in scleral surgery methods for presbyopia correction [217,226,227].

Currently, the only scleral implant available on the market potentially intended for presbyopia correction and analyzed by the FDA is the VisAbility Micro-Insert scleral implant [228].

During the VisAbility Micro-Insert implantation procedure, four PMMA implants are injected into the sclera approximately 3000–4000 µm from the limbus at a depth of 400 µm. By elevating the sclera and ciliary body, the implants increase zonular tension and help restore the eye’s lost accommodative amplitude [107,228,229].

In 2013, results from a study evaluating the effectiveness of the VisAbility Micro-Insert in presbyopia correction were presented [230]. 73% of treated patients were satisfied with the achieved visual acuity, whereof 99% satisfied with distance vision and 76% satisfied with near vision [230].

The procedure provided independence from near vision correction glasses in 83% of patients [230].

Complications that may occur after the procedure include: anterior segment ischemia, conjunctival erosions, subconjunctival hemorrhages, infections and endophthalmitis [230].

Additionally, according to FDA reports, 75% of patients treated with the first-generation VisAbility Micro-Insert experienced implant displacement [231].

Due to the occurrence of serious adverse effects such as anterior segment ischemia in the above procedures, methods with higher safety profiles were sought. This led to the development of scleral laser excision (SLE), first performed by Lin et al. in 1998 [232,233].

During the SLE procedure, nearly full-thickness scleral ablation was performed [233].

A specific SLE technique aimed at presbyopia correction was laser presbyopia reversal (LAPR).

In LAPR procedures, an erbium:YAG laser was used to perform a series of scleral resections at a depth of 500–600 µm, approximately 4500 µm in length and 600–700 µm in width [233].

Twelve months after the procedure, an increase in recovered accommodative amplitude by 2 D was observed [233].

Despite its demonstrated effectiveness, this therapeutic option is nowadays not in use [234].

The only currently available scleral laser surgery procedure used in presbyopia correction is laser anterior ciliary scleral excision (Laser ACE) [224].

This procedure is not based on Schachar’s theory but rather fulfills the assumptions of the dynamic theory of presbyopia development [27,235].

The effectiveness of Laser ACE is thought to result not from geometric changes within the sclera and ciliary muscle, but from a modification of the ocular tissues aimed at improving the efficiency of the structures involved in the accommodation process [27,31].

During the procedure micropores about 600 µm wide and 500–700 µm deep are made in the sclera using an erbium: YAG laser. These micropores are made in the four quadrants of the eyeball above three anatomically and physiologically critical zones: 0.5–1.1 mm, 1.1–4.9 mm and 4.9–5.5 mm from the anatomical limbus [27,31,236].

This results in the formation of scleral zones with differing stiffness, which modulates the eye’s mechanical response when ciliary muscle contracts and improves the function of accommodative structures [27,31,236].

In 2017, the results of a 24-month study evaluating the efficacy of Laser ACE in presbyopia correction were published [237]. These results presented by the authors were as follows:

- monocular UNVA improved from 0.36 ± 0.2 logMAR before the procedure to 0.25 ± 0.18 logMAR after the procedure,
- binocular DCNVA improved from 0.21 ± 0.17 logMAR before the procedure to 0.11 ± 0.12 logMAR after the procedure,
- a DCNVA equal to or exceeding 0.2 log MAR was reached by 83% patients,
- stereopsis improved from 75.8 ± 29.3 arcseconds before the procedure to 58.8 ± 22.9 arcseconds after the procedure [237].

Adverse effects of the procedure may include accidental scleral microperforation and subconjunctival hemorrhages [237].

The effectiveness of scleral surgery methods based on Schachar’s theory assumptions in the correction of presbyopia largely depends on the patient’s age.

Based on data obtained using magnetic resonance imaging, it has been determined that the ciliary body-lens diameter difference decreases by about 0.02–0.025 mm each year [238,239]. Scleral surgery techniques, can lead to 0.1 mm enlargement of ciliary body diameter which in turn allows recovery of the functional mobility of accommodative structures to levels seen 4–5 years prior [33,238,239]. This limitation may be bypassed by the LaserACE technique.

### 5.2. Refractive Lens Exchange in Presbyopia Correction with Implantation of an Accommodating Intraocular Lens

Refractive lens exchange (RLE) with implantation of an accommodating intraocular lens (AIOL) represents a surgical strategy designed to restore physiologic accommodation by enabling dynamic changes in optical power in response to ciliary muscle contraction. Unlike multifocal or extended-depth-of-focus (EDOF) intraocular lenses, AIOLs aim to provide presbyopia correction without compromising image quality, relying on biomimetic movement or deformation of the optic rather than simultaneous vision or diffractive optics. Contemporary AIOLs can be divided according to their optical and biomechanical design into single-optic, dual-optic, and deformable-optic systems [240].

Single-Optic Accommodating IOLs

Single-Optic AIOLs rely on the transmission of ciliary muscle forces to the capsular bag and lens haptics, inducing anterior axial displacement, and subtle optic deformation that increase near refractive power. Representative designs include the 1CU, Tetraflex KH3500, Crystalens^®^ AT-45, and Crystalens^®^ HD, with the Crystalens family being the only models that have achieved U.S. FDA approval [240].

- Crystalens^®^ AT-45

The Crystalens AT-45 is a three-piece silicone posterior-chamber lens with a 4.5 mm optic, designed to vault anteriorly under accommodative effort. Objective measurements demonstrate accommodative amplitudes of approximately 0.44–1.0 D, reflecting the limited true axial shift achievable in vivo [241,242]. Importantly, contrast sensitivity remains comparable to monofocal IOLs, preserving high-quality distance vision. However, optical side effects have been reported, including optic tremor, which may induce spherical aberration and astigmatism. Posterior capsular opacification (PCO) represents a significant limitation, occurring at higher rates than with monofocal lenses [243,244,245].

- Crystalens^®^ HD

The Crystalens HD incorporates a modified optic profile to enhance intermediate and near visual acuity. In vivo measurements report axial shifts of up to 1.4 mm, with clinical studies noting superiority in intermediate and near vision relative to monofocal lenses. Nevertheless, PCO remains prevalent, affecting up to 40.7% of implanted eyes [240,246,247].

Limitations of Single-Optic AIOLs

Despite their conceptual advantages, single-optic AIOLs demonstrate:

- Low objective accommodative amplitudes (<0.4–1.0 D), often declining over time,
- Progressive loss of mobility due to capsular fibrosis and stiffening,
- Higher-than-monofocal rates of PCO, although YAG capsulotomy generally does not impair lens mobility [241,242,243,244,245,248].

Long-term outcomes indicate that single-optic translational mechanisms are significantly restricted by postoperative capsular changes, limiting their ability to maintain durable accommodation [240].

Dual-Optic Accommodating IOLs

Dual-optic AIOLs incorporate two interconnected optics: an anterior high-positive lens and a posterior negative lens. During accommodation, changes in ciliary muscle tension alter the spacing between the two optics, increasing the combined optical power. This design enhances the accommodative effect while requiring small physical displacements [240,249].

Dual-optic systems have demonstrated substantially higher accommodative amplitudes (~3–4 D) than single-optic lenses in clinical studies [249,250,251].

Additional advantages include:

- Reduced dependence on optic axial shift [252],
- Lower rates of PCO compared with single-optic designs [240,246],
- Sustained early postoperative near vision performance [240].

Limitations

Despite their superior optical potential, dual-optic lenses face specific challenges:

- Image magnification secondary to anteriorly positioned high-plus optics [253],
- Performance variability depending on axial length [254],
- Decline in accommodative amplitude over time due to capsular fibrosis and shrink-wrap phenomena, which restrict optic separation [242].

Prototypes such as the Synchrony lens demonstrated feasibility for implantation through small incisions (3.8–4.0 mm), but long-term outcomes have remained constrained by the biological limitations of the capsular bag [249].

Deformable-Optic Accommodating IOLs

Deformable AIOLs represent a newer generation of designs aiming to reproduce the natural changes in lens curvature. These lenses typically consist of a flexible optical shell filled with fluid or gel-like material. Ciliary muscle contraction deforms the shell and redistributes internal fluid, generating dynamic and potentially larger changes in refractive power [240,255].

Early investigations have indicated accommodative amplitudes up to 10 D, though such results remain preliminary and dependent on ex vivo or early-phase clinical data. The underlying concept offers the advantage of curvature change—more closely mimicking the physiologic lens, rather than relying solely on axial movement [255].

Emerging Next-Generation AIOL Technologies

Several advanced AIOL platforms have re-emerged in recent years as potential competitors to monofocal, multifocal and EDOF IOLs.

These include:

- FluidVision (PowerVision/Alcon)

The FluidVision accommodative intraocular lens (IOL) incorporates a hydrophobic acrylic shell filled with silicone oil. Contraction of the ciliary muscle drives fluid toward the central optic, modifying its curvature and increasing optical power in a manner intended to replicate physiologic accommodation. Clinical reports generally indicate an accommodative amplitude of approximately 2.0 D, with some eyes demonstrating up to 4.1 D under optimal testing conditions [256,257]. In a multicenter pilot study of monocular implantation, best-corrected distance visual acuity (BCDVA) remained stable at six months (−0.05 logMAR). At the same interval, distance-corrected intermediate visual acuity averaged ~0.05 logMAR (≈20/22) and near visual acuity ~0.14 logMAR (≈20/27) [256].

Additional evidence is provided by a single-site study conducted by Dr. Potgieter and colleagues, enrolling 26 patients and evaluating accommodative function, visual quality and long-term stability through 24 months. Eight patients received a fellow-eye implant 15–21 months after their initial surgery. Among the seven patients with available binocular data at one month, mean best distance-corrected visual acuities were: 0.05 ± 0.05 logMAR at distance, 0.05 ± 0.06 logMAR at 66 cm and 0.16 ± 0.16 logMAR at 40 cm. Binocular intermediate and near acuities were approximately one line better than corresponding monocular values. Mean monocular uncorrected distance visual acuity was −0.01 logMAR (20/20+) and binocular uncorrected distance acuity reached 0.00 logMAR (20/20). Defocus-based accommodative amplitudes averaged 3.12 D monocularly and 4.19 D binocularly [256].

According to Dr. Potgieter, the FluidVision^®^ IOL provides excellent binocular acuity from distance to near and is distinguished by its continuous range of focus, rather than the discrete focal points characteristic of diffractive multifocal designs. Contrast sensitivity exceeded that of standard diffractive multifocal IOLs under mesopic and mesopic-glare conditions and was comparable to that of monofocal IOLs. Stability and safety were maintained through 24 months, with endothelial cell counts remaining within expected postoperative limits [256].

Although the FluidVision accommodative IOL has generated considerable interest due to its fluid-driven shape-changing design, it is important to emphasize that most currently available clinical data originate from conference presentations, industry reports, and non-peer-reviewed sources. Evidence from a single-site prospective series reported by Potgieter and colleagues suggests promising visual outcomes, including binocular defocus-based accommodative amplitudes exceeding 4.0 D and stable best-corrected distance visual acuity through 24 months. However, these findings have been disseminated primarily through scientific meeting abstracts and ophthalmic trade publications rather than full-length, peer-reviewed manuscripts. Similarly, early multicenter pilot results frequently cited in the literature—including accommodative amplitudes of approximately 2–4 D and preservation of contrast sensitivity comparable to monofocal IOLs, are derived from congress communications and manufacturer-sponsored reports, which limits the ability to independently assess methodology, statistical rigor, and long-term consistency. To date, no large-scale, randomized, peer-reviewed clinical trials have been published that validate these outcomes or address potential long-term issues such as capsular fibrosis, mechanical fatigue of the fluid-filled system, or durability of accommodative performance. Consequently, while early data regarding the FluidVision IOL are encouraging, they should be interpreted cautiously until corroborated by robust, independently verified, peer-reviewed clinical studies.

- Juvene (LensGen)

The Juvene accommodative intraocular lens (IOL) represents one of the most ambitious attempts to recreate natural accommodation within an artificial lens system. Its design is notably modular: a stable base lens sits securely in the capsular bag, while a separate fluid-filled optic attaches anteriorly to provide the dynamic, shape-changing component. When the eye engages in the accommodative reflex, the capsular bag transmits subtle mechanical forces to the optic, deforming the fluid chamber and altering the curvature of the lens. This shift in shape changes the refractive power in a manner that more closely resembles natural accommodation than the multifocal or extended depth-of-focus strategies typically employed today [258].

Early clinical insights came from the GRAIL study, which followed patients for 12 months after Juvene implantation. The visual acuity outcomes were encouraging: monocular CDVA reached 0.01 logMAR, with intermediate and near performance also demonstrating functional acuity (0.08 and 0.24 logMAR, respectively). When tested binocularly, results improved further, reflecting the expected summation benefits. Importantly, patients exhibited stable refraction in the early postoperative period, with no meaningful shift in spherical equivalent during the first three months—an indicator of mechanical and positional stability within the capsular bag [259].

Since 2021, clinical trials in the United States have expanded the evidence base, leading to the release of three-year results presented at the 2023 ASCRS meeting. At 36 months, the mean monocular acuities for far, intermediate and near distances were 20/18, 20/26, and 20/35. These values not only reflect impressive distance clarity but also a functional range of intermediate and near vision without reliance on diffractive optics. The accommodative response, too, has been a point of interest. Defocus data at six months suggested approximately 2.5 D of monocular accommodation, with bilateral implantation achieving around 3.0 D. In a subset of the study population, more robust amplitudes of up to 3.5 D were documented, hinting at the device’s potential when all mechanical factors align optimally. Reading performance and speed corresponded well to these findings, supporting the idea that the Juvene lens can provide truly usable accommodation rather than merely modest depth-of-focus extension [260].

Safety outcomes have been similarly reassuring. Mesopic contrast sensitivity, a common weakness among multifocal IOLs, has been repeatedly reported as comparable to monofocal lenses, with minimal glare or halo complaints [259]. The mechanical stability of the system appears to hold over time: effective lens position and rotation remained consistent through 36 months, endothelial cell counts were preserved and even after four years of follow-up in some patients, posterior capsule opacification had not emerged as a significant issue [260].

Taken together, the evidence portrays the Juvene system as a maturing technology with genuine promise. Its biomimetic, fluid-based mechanism offers an alternative to traditional multifocality, aiming instead to restore a more natural visual experience across multiple distances. With ongoing studies and expanding long-term data, the lens continues to position itself as a leading candidate in the next generation of accommodative IOLs.

- Lumina (AkkoLens)

The Lumina AIOL represents a distinctive approach to restoring functional near vision by harnessing ciliary-body forces in a sulcus-fixated design. Unlike capsular bag-based accommodative IOLs, the Lumina is engineered specifically for placement in the ciliary sulcus, where it maintains direct contact with the ciliary body. Its structure consists of two hydrophilic acrylic refractive elements arranged as flexible omega-loops that behave like springs, supported by rigid connectors that secure the system to a central lens base. When the ciliary body contracts, these elements translate perpendicularly to the optical axis, gradually modulating the effective refractive power of the lens. Because the lens must sit optimally within the sulcus, each Lumina implant is custom-sized according to the individual eye’s sulcus-to-sulcus measurement [258,261].

Clinical evaluation of the Lumina has unfolded through several important studies. One of the earliest and most influential was a randomized clinical trial comparing visual and accommodative outcomes between the Lumina and a standard monofocal IOL (AcrySof SA60AT). The trial included 86 eyes undergoing cataract surgery, with 61 receiving the Lumina lens and 25 receiving the monofocal control lens. Over the 12-month follow-up, both uncorrected and corrected distance visual acuities remained statistically similar between groups. However, near performance was markedly different. The Lumina group achieved substantially better uncorrected near visual acuity (0.07 ± 0.08 logRAD) compared to the monofocal controls (0.37 ± 0.19 logRAD) and the same pattern was observed for corrected near results (0.11 ± 0.12 vs. 0.41 ± 0.15 logRAD). Defocus curve analysis revealed significantly superior performance for the Lumina from −4.50 to −0.50 D, reflecting enhanced functional near range [258,262].

Accommodation was measured both subjectively—via defocus curves, and objectively using an open-field autorefractor. Subjective accommodation clearly favored the Lumina, which demonstrated amplitudes of 3.05, 3.87, and 5.59 D at visual acuities of 0.10, 0.20, and 0.40 logMAR, respectively. In contrast, the monofocal control lens provided only 1.46, 2.00, and 3.67 D at those same thresholds. Objective accommodation demonstrated a similar trend: the Lumina produced measurable accommodative changes ranging from 0.63 to 1.27 D across accommodative stimuli of 2.0–4.0 D, while the monofocal lens exhibited minimal or no objective response. Importantly, contrast sensitivity remained comparable between groups, supporting the lens’s safety in terms of optical quality [263].

More recently, the evolution of Lumina technology has continued, culminating in a late-stage, prospective bilateral implantation study published in 2025. Conducted at the Cornea, Cataract, and Refractive Surgery Unit of Vissum, Grupo Miranza (Alicante, Spain), this longitudinal study followed 25 patients for 12 months after receiving bilateral Lumina IOLs. The results reinforced the early randomized trial’s findings while adding new insights into optical quality and patient-reported outcomes. Distance and near acuities improved significantly postoperatively, with mean uncorrected values of 0.06 ± 0.15 logMAR at distance and 0.27 ± 0.15 logMAR at near. The defocus curve again illustrated the lens’s extended functional range, with corrected vision of 0.01 ± 0.06 logMAR for distance, 0.18 ± 0.11 for intermediate and 0.38 ± 0.13 for near [264].

Subjective depth of focus reached 1.37, 2.05, and 3.63 D for acuity thresholds of 0.10, 0.20, and 0.40 logMAR. Objective accommodation averaged −0.65 ± 0.69 D, a small but measurable improvement consistent with the lens’s translational mechanism. Notably, contrast sensitivity exceeded normal reference values and optical quality metrics such as the point spread function (mean 0.23 μm) indicated a well-preserved retinal image. Patient-reported outcomes were particularly positive: over 87% of participants described mild or no difficulty with uncorrected near vision and most reported good functional performance at both near and far distances with minimal dysphotopsias [263].

Safety outcomes across studies have been favorable. Complications were limited primarily to posterior capsule opacification, which—when present, responded well to laser capsulotomy and was associated with improved near acuity thereafter. No significant issues with glare, halos, or contrast loss have been observed, which distinguishes the Lumina from multifocal IOLs that often compromise optical quality to achieve extended range of focus [262,263,264].

Taken together, the growing body of evidence positions the Lumina lens as an innovative sulcus-fixated accommodative IOL capable of providing enhanced near vision and measurable accommodative response while maintaining the optical clarity characteristic of monofocal lenses. As long-term data continue to expand, the Lumina stands out as a compelling direction in the pursuit of physiologic, high-quality pseudophakic accommodation.

- OmniVu (Atia Vision)

The OmniVu Atia Vision lens is a next-generation accommodative intraocular lens (IOL) built around a segmented, dual-optic design. Its fluid-filled base sits in direct contact with the capsular bag, allowing ciliary muscle activity to induce shape changes that modulate the lens’s refractive power. A separate anterior static optic does not mechanically interact with the base but refines the overall refractive outcome, creating a modular system intended to deliver smooth, dynamic focusing across distances [265].

Early first-in-human results, presented at professional meetings, suggest promising visual performance. At six months post-implantation, monocular uncorrected distance vision reached about 20/20, with intermediate and near acuities around 20/25 and 20/32. Refractive predictability was high, with 95% of eyes within ±0.50 D of plano. Bilateral implantation appeared to enhance results further, producing binocular vision of 20/16 at distance, 20/20 at intermediate and 20/32 at near. Although these findings indicate meaningful accommodative potential, they remain preliminary, as no peer-reviewed long-term data have yet been published [266].

These early findings, while informative, have not yet undergone formal peer review or been published in full-length, independently validated clinical studies. As such, the results should be interpreted with appropriate caution until corroborated by rigorous, peer-reviewed clinical trials.

A key development occurred in May 2025, when the U.S. FDA granted Investigational Device Exemption approval for the OmniVu, authorizing the start of formal clinical trials in cataract patients. With this step, the lens moves from early feasibility into controlled clinical evaluation. As the trials progress, more definitive evidence will clarify whether the OmniVu design can deliver reliable, physiologic accommodation and establish itself among emerging accommodative IOL technologies [267].

- Opira lens

The Opira AIOL (ForSight Vision6) represents a novel sulcus-fixated, dual-element design aimed at restoring functional accommodation through direct ciliary body interaction. The device consists of two independently implanted components: a static posterior lens and a dynamic anterior deformable optic. The anterior element is designed to alter its curvature in response to ciliary muscle contraction, thereby increasing refractive power without relying on capsular bag integrity. Because the haptics of the Opira IOL anchor to the capsulorrhexis edge, surgical success depends on a precisely sized and centered capsulotomy. For this reason, the lens may benefit from femtosecond laser capsulotomy or precision-pulse systems such as Zepto (Centricity Vision) to optimize support and alignment [260].

Early clinical evaluation has been limited but suggests encouraging visual performance. A preliminary 16-patient clinical study (non–peer-reviewed) reported uncorrected near visual acuity equivalent to 20/25 following Opira implantation. In vitro optical testing demonstrated monofocal-quality image performance across the full defocus range, supporting the lens’s ability to deliver accommodation without the contrast trade-offs typical of multifocal optics [268].

Two-year follow-up data from a non-peer-reviewed cohort study involving 29 patients who received a monofocal IOL in one eye and the Opira lens in the contralateral eye, showed clinically meaningful advantages for the Opira-implanted eyes. Distance-corrected intermediate and near visual acuities were superior with the Opira lens, consistent with its intended accommodative function. Posterior capsule opacification occurred frequently, but Nd: YAG laser capsulotomy improved visual quality and did not appear to compromise accommodative amplitude. Importantly, no cases of uveitis-glaucoma-hyphema (UGH) syndrome have been reported, a finding attributed to the mechanical stability and smooth surface profile of the device. Manufacturer reports indicate that the Opira lens can be inserted through a 3.9 mm clear corneal incision, with ongoing design refinements expected to further reduce incision size [260,269].

Additional non-peer-reviewed data presented by David Chang, MD, at Hawaiian Eye 2024, summarized outcomes for 32 patients implanted with the Opira lens. These findings indicated excellent uncorrected visual acuities at distance, intermediate and near, with 97% of patients reporting spectacle independence [270]. Patient-reported outcomes also suggested reduced rates of glare and halos compared with both monofocal and trifocal IOLs [260,270]. Across available datasets, no instances of IOL dislocation or explantation have been documented [269].

Although the Opira IOL embodies an innovative approach to achieving physiological accommodation—particularly through its capsulorrhexis-fixated, sulcus-positioned design, the current evidence base remains composed largely of early-phase, non-peer-reviewed data and manufacturer-reported outcomes. Rigorous, peer-reviewed clinical trials will be essential to confirm long-term stability, accommodative performance and safety relative to existing presbyopia-correcting IOL technologies.

Accommodating intraocular lenses continue to evolve toward the goal of restoring true, physiologic accommodation after lens removal. While single-optic designs provide good image quality, their accommodative amplitudes remain small and decline with capsular fibrosis. Dual-optic lenses achieve higher early accommodative amplitudes (~3–4 D), but similarly face challenges related to long-term capsular changes. Deformable-optic designs, including fluid-based and curvature-changing systems, represent the most promising frontier, with the potential for significantly greater dynamic power change and reduced reliance on capsular bag biomechanics.

Future progress will depend on:

- Minimizing the impact of capsular fibrosis and shrink-wrap,
- Improving mechanical stability and long-term predictability,
- Verifying accommodative performance with objective metrics,
- Conducting large-scale, controlled, long-term clinical trials.

Although current AIOLs do not yet fully replicate youthful accommodation, next-generation deformable and hybrid systems offer a tangible pathway toward achieving functional, dynamic presbyopia correction with uncompromised optical quality.

Table 3 summarizes the discussed above presbyopia correction methods [33].

## 6. Conclusions

Presbyopia management is rapidly evolving, driven by advances in optical engineering, biomimetic design and pharmacological innovation. Several key take-home messages emerge from the current evidence. No single modality fully restores youthful accommodation, but incremental improvements across corneal, lenticular, scleral and pharmacologic strategies are steadily enhancing functional outcomes. Light-adjustable intraocular lenses have markedly improved postoperative refractive predictability, while next-generation accommodating IOLs aim to reproduce true dynamic changes in lens shape rather than relying solely on monovision or diffractive multifocality.

Laser-based corneal refractive procedures remain an important component of presbyopia correction, particularly presbyLASIK, blended vision strategies and procedures inducing controlled multifocality or spherical aberration adjustments. While these techniques can offer good visual performance and rapid rehabilitation, their ability to correct presbyopia is inherently limited in range and durability. Because they do not restore accommodation but instead reshape the cornea to enhance pseudoaccommodation, their effectiveness typically declines over time as the crystalline lens continues to stiffen and the amplitude of accommodation further diminishes. Consequently, they cannot guarantee lifelong efficacy and many patients may require complementary treatments or enhancement procedures in later years.

Extended depth-of-focus and full range of vision IOLs significantly broaden the spectrum of surgical presbyopia correction. EDOF optics offer continuous distance-to-intermediate vision with fewer photic phenomena than diffractive multifocal IOLs, though near vision remains limited in many designs. Full range of vision lenses extend this concept by enhancing intermediate and functional near performance, providing smoother defocus curves and greater spectacle independence. Nevertheless, mild photic disturbances may still occur and clinical results can vary depending on neural adaptation.

Despite these advances, major unmet needs persist, including: achieving stable, reproducible true accommodation, preventing postoperative capsular fibrosis that restricts lens movement, establishing standardized metrics to quantify accommodative performance and developing solutions with minimal optical compromises. Reversibility, adjustability, long-term biocompatibility and consistent visual performance across lighting conditions remain critical goals.

The therapeutic landscape is further expanding with emerging pharmacological approaches. Beyond miotic agents that enhance depth of focus, investigational therapies targeting crystalline lens biomechanics, protein aggregation or ciliary muscle physiology may help preserve or partially restore accommodation. Such treatments may ultimately complement surgical strategies or offer non-invasive alternatives for individuals seeking temporary or staged presbyopia management.

Looking ahead, the most promising technologies are those that combine high-quality optical performance with mechanisms closely mimicking physiological accommodation. Biomimetic deformable-optic IOLs, including devices such as Juvene, Lumina, FluidVision, OmniVu and Opira, represent a significant shift toward restoring genuine accommodative amplitude and may play a central role in the next generation of presbyopia correction. Corneal laser surgery is likely to evolve into a supportive modality, offering selective enhancement rather than definitive correction, while pharmacologic innovations may provide personalized or adjunctive solutions across the presbyopic lifespan. Collectively, while complete restoration of youthful accommodation remains one of the greatest challenges in anterior segment surgery, the convergence of lenticular, corneal, scleral and pharmacological strategies suggests that achieving stable, functional, physiologic accommodation may become a realistic clinical objective within the next decade.

This review has several inherent limitations. First, although multiple sources were consulted, the search was not conducted using a predefined protocol, formal inclusion and exclusion criteria, or a systematic review framework. As a result, the selection of studies may reflect an element of narrative bias. Second, the review draws on a combination of peer-reviewed publications, conference communications and selected press or industry reports, which may differ in methodological rigor and level of evidence. Third, the rapidly evolving nature of presbyopia research means that some emerging technologies may not yet be represented in high-quality clinical trials, limiting the ability to compare them directly. Finally, heterogeneity among available studies—differences in endpoints, follow-up duration, patient populations and outcome measures—restricts the possibility of synthesizing results quantitatively. These limitations should be considered when interpreting the conclusions of this narrative overview.

## Figures and Tables

**Table 1:** Summary of the characteristics, mechanisms and clinical outcomes of intracorneal implants [86,87,88,89,90,91,92,93,94,95,96,97,98,99,100,101,102,103,104,105].

Inlay	Type	Implantation Depth/Method	Mechanism of Action	Near Vision Outcome	Distance Vision Outcome	Complications/Safety Issues
Presbia Flexivue Microlens	Refractive	300 µm corneal pocket, non-dominant eye	Bifocal structure: central distance zone, peripheral near-vision zone (+1.25 to +3.5 D)	UNVA improved; one or more lines of CDVA reduction in 60%; >3-line loss in 10%	Minor decrease in CDVA in some patients	CDVA loss, potential chronic keratocyte irritation, foreign body response
Raindrop Near Vision Inlay	Corneal reshaping	200 µm in corneal stroma or under LASIK flap, non-dominant eye	Hyperprolate anterior corneal curvature -multifocal cornea	Near and intermedi-ate vision improved	Distance vision generally maintained	Corneal haze (up to 42% after 5 years), explantation in 7–12%, FDA withdrawal 2018
KAMRA Inlay	Small-aperture	200–250 µm in corneal stroma, non-dominant eye	Pinhole effect—increased depth of focus	Near and intermediate vision improved	Minimal impact on distance vision	CDVA loss ≥ 2 lines in 3.4%, corneal haze/explantation 8%, hyperopic shift, binocular asymmetry
TransForm Corneal Allograft (TCA)	Allogenic	Femtosecond laser pocket along pupil axis, SMILE-derived lenticule	Native tissue implantation, refractive modification without synthetic material	UNVA improved from 0.52 ± 0.14 to 0.20 logMAR at 6 months	BCDVA mostly unchanged 1–2 line reduction in few cases	Small risk of graft rejection, centration issues, limited sample size
PEARL (Presbyopic Allogenic Refractive Lenticule)	Allogenic	120 µm intracorneal pocket via femtosecond laser, SMILE-derived lenticule	Native tissue implantation, refractive modification without synthetic material	UNVA J2 achieved in all eyes	UDVA 20/20 in all eyes	Limited data, potential graft rejection and centration challenges

**Table 2:** Comparative Clinical Performance of PresbyMAX and Presbyond.

Category	PresbyMAX (SCHWIND)	Presbyond (Carl Zeiss)
Optical principle	Bi-aspheric multifocal ablation, central hyperpositive near-add zone (+0.75 to +2.50 D), smooth aspheric transition, reduced spherical aberration.	Non-linear aspheric ablation, controlled spherical aberration induction, micro-monovision (−0.75 to −1.5 D), blended vision zone.
Algorithmic modes	Symmetric, Hybrid, µ-Monovision, customizable distance/near balance.	Single personalized algorithm, ablation individualized by age, aberration, anisometropia tolerance.
UDVA outcomes	70–83% ≥ 20/25, up to 93% in micro-monovision [136,137,138,139].	98–99% binocular 20/20 in myopes, 98% in emmetropes, 90% in hyperopes [142,143,144].
UNVA outcomes	80–90% reach 0.1–0.3 logRAD, 90% J2 in micro-monovision [136,137,138,139].	96% J2 or better in emmetropes, J5 in myopes/hyperopes [142,143,144].
UIVA outcomes	Very good, up to 97% J2 [137].	Excellent intermediate due to extended depth of field [142,143].
Loss of CDVA	Up to 10% lose ≥ 2 lines depending on refractive group, 7% in micro-monovision [136,137,139].	None lose > 2 lines across studies [142,144].
Retreatment/enhancement	10–19% within 12 months [125,133,134,136,139].	Very low, enhancements uncommon [142,146].
Stability (12–36 months)	High stability, mild hyperopic drift in hyperopes long-term [125,134,135,136,139].	Highly stable, refractive shift −0.06 to +0.36 D [142,143,144,145,146].
Contrast sensitivity	Generally preserved, mild scotopic reduction [134,140].	Not impaired across groups [119,140].
Night-vision disturbances	Mild early glare/halos resolving by 3–6 months [125,133,134,139].	Very low, rare mild dysphotopsia (6.6%) [156].
Stereopsis	Mostly preserved, may reduce depending on monovision level [140].	Mostly preserved, temporary reduction possible, improves by 6 months [139,140,147,148,149,150,151,152,153,154,155].
Patient satisfaction	High (>90%), strongest in hyperopes [136,139].	Very high (93–100% across distances) [146,156].
Best candidates	Myopes, hyperopes, emmetropes requiring customizable multifocality.	Adults > 40; myopia ≤ −8 D, hyperopia ≤ +4 D, good monovision tolerance.
Distinct advantages	Customizable algorithms, strong intermediate vision.	Superior binocular integration, minimal CDVA loss, excellent night-vision profile.
Key limitations	Higher retreatment, possible line loss, temporary aberration increase.	Temporary stereopsis reduction, dryness/fluctuations early.

**Table 3:** Summary of discussed presbyopia correction methods [33].

Static Methods					Dynamic Methods	
Conservative Methods			Surgical Methods			
Optical Aids	Pharmacotherapy					
Spectacles	Muscarinic receptor agonists	Pilocarpine	Corneal inlays synthetic and allogenic		Scleral surgery	Anterior Ciliary Sclerotomy
		Carbachol				Scleral Expansion Bands
		Aceclidine				VisAbility Micro-Insert scleral implant
						Scleral Laser Excision
						Laser Anterior Ciliary Scleral Excision
Contact lenses	Non-selective adrenergic receptor blocker	Phentolamine	Conductive keratoplasty		Refractive lens exchange with implantation of accommodating intraocular lense	
	Combined medications	Pilocarpine and phenylephrine	Corneal laser refractive surgery	Laser induced monovision		
		Pilocarpine and diclofenac		Multifocal ablation		
		Carbachol and brimonidine		Non-linear aspheric ablation profile with micro-monovision (Presbyond LBV)		
	Drugs reducing lens stiffness		Refractive lens exchange	Monofocal IOLs		
				Monofocal plus IOLs		
				EDOF and FRoF IOLs		
				LALs and LALs+		

## Data Availability

No new date were created or analyzed in this study. Data sharing is not applicable to this article.
